# Effects of musculoskeletal expert input on low back pain outcomes: A randomised controlled trial

**DOI:** 10.1371/journal.pone.0356604

**Published:** 2026-08-26

**Authors:** Janny Mathieu, Marie Beauséjour, Wanda Dupèbe, Claude-Édouard Châtillon, Julie O’Shaughnessy, Charles Tétreau, Cesar A. Hincapié, Petra Schweinhardt, Martin Descarreaux, Andrée-Anne Marchand

**Affiliations:** 1 Department of Anatomy, Université du Québec à Trois-Rivières, Trois-Rivières, Quebec, Canada; 2 Department of Community Health Sciences, Université de Sherbrooke, Longueuil, Quebec, Canada; 3 Department of Medical Biology, Université du Québec à Trois-Rivières, Trois-Rivières, Quebec, Canada; 4 Centre intégré universitaire de Santé et de Services Sociaux de la Mauricie-et-du-Centre-du-Québec, Trois-Rivières, Quebec, Canada; 5 Division of Neurosurgery, Faculty of Medecine, Université de Montréal, Montréal, Quebec, Canada; 6 Chiropractic Department, Université du Québec à Trois-Rivières, Trois-Rivières, Quebec, Canada; 7 Department of Human Kinetics, Université du Québec à Trois-Rivières, Trois-Rivières, Quebec, Canada; 8 Epidemiology, Biostatistics and Prevention Institute (EBPI), University of Zurich, Zurich, Switzerland; 9 University Spine Centre Zurich (UWZH), Balgrist University Hospital, Zurich, Switzerland; 10 Dalla Lana School of Public Health, University of Toronto, Toronto, Canada; 11 Department of Chiropractic Medicine, Balgrist University Hospital, Zurich, Switzerland; University Hospital Zurich, SWITZERLAND

## Abstract

**Background:**

Overuse of low-value interventions and delays in accessing appropriate care contribute to the growing burden of low back pain (LBP). Integrating musculoskeletal health experts into care pathways may represent a pragmatic strategy to improve access, referral appropriateness, and efficiency of spine care delivery.

**Purpose:**

This study evaluated the effects of an evidence-based triage assessment performed by chiropractors on clinical outcomes and care trajectories of patients with LBP referred for a neurosurgical consultation. We hypothesised that the intervention would produce equivalent clinical outcomes while improving care trajectories.

**Study design:**

Single-center, parallel-group randomised controlled trial

**Outcome measures:**

Patient reported measures were collected at 1 week, 3, 6 and 12 months, including back pain intensity (primary outcome), leg pain intensity, LBP-related disability, quality-of-life, perception of change, and care trajectories.

**Methods:**

One hundred and one adults with LBP were randomized to the intervention (n = 52) or control group (n = 49). All participants underwent a standardized chiropractor-led triage evaluation prior to the neurosurgical consultation. In the intervention group, the chiropractor’s assessment findings and recommendations were communicated to the neurosurgeon before the consultation, whereas they were not shared for the control group. Equivalence for the primary outcome (back pain intensity) was assessed at the 3-month primary endpoint using the two one-sided tests procedure. Repeated-measures ANOVAs examined changes in patient-reported clinical outcomes (i.e., back pain and leg pain intensity, LBP-related disability, quality of life and perception of change), while mixed-effects regression models were used to compared care trajectories outcomes between groups.

**Results:**

Although equivalence was not statistically demonstrated as the confidence interval extended beyond the predefined equivalence margins of ± 1.2 points on the NRS-11, between-group differences in back pain intensity remained small and not clinically meaningful (0.44 ± 0.54 [95% CI −1.51-0.63]). No significant between-group differences were observed across clinical or care trajectories outcomes, except that, fewer participants in the intervention group reported consulting a general practitioner at 6 months (49.0% vs. 69.2%; p = 0.04).

**Conclusion:**

These findings suggest that integrating chiropractors into the triage of LBP referrals does not adversely affect clinical outcomes and may influence care trajectories. Although most care trajectory outcomes were similar between groups, participants in the intervention group reported fewer general practitioner consultations at 6 months. Future research should investigate triage strategies implemented in primary care settings, where their potential to optimize patient trajectories may be greater.

## Background

Underutilization of evidence-based practices, including both overuse of low-value care approaches (e.g., routine diagnostic imaging, opioids, spinal injections, and surgery) and insufficient implementation of high-value care interventions (i.e., patient education, physical activity, and self-management support) [1] are identified as major contributors to the growing burden of low back pain (LBP) [2]. A systematic review and meta-analysis of 21 studies from 12 high-income or middle-income countries estimated the prevalence of consultations to emergency departments (ED) for LBP to be 4.39% (95% CI 3.67–5.18), similar to that for shortness of breath, fever, and chills [3]. In line with these findings, a prospective observational study of patients with LBP attending the ED of a university hospital in Edmonton, Canada, also revealed that most common reasons for seeking care were an inability to control pain and a need to clarify the etiology of symptoms [4]. In most cases, patients were discharged after nurse triage without a physician consultation (89.0%) [4]. Interestingly, when asked which clinician they would prefer to manage their LBP, assuming no financial or accessibility constraints, 42.6% of participants still indicated a preference for ED physicians [4]. All together, these observations suggest that EDs are often not the most appropriate care setting for patients with LBP and underscore the need for patient education, reassurance, and care pathways that engage musculoskeletal health professionals in delivering evidence-based interventions.

The 2018 Lancet Series identified promising directions for the prevention and management of LBP, including the redesign of clinical pathways to enable people to have access to appropriate healthcare resources in a timely manner, while avoiding exposure to ineffective and potentially unsafe treatments [2,5]. In recent years, several models of care and clinical pathways have been proposed in high-income countries, aiming to achieve better health outcomes, improved care experience, and more efficient use of healthcare resources for patients with LBP [1]. In a scoping review including 29 studies from multiple countries, Duarte et al. (2024) [6] identified 11 care models implemented in primary care for LBP. General practitioners served as an entry point for all but 3 models of care, and all but one model included interface services (i.e., services that incorporate any intermediate levels of triage, assessment, and treatment between primary care and secondary care), mostly operated by multidisciplinary teams [7]. In Canada, three models of care have been developed: the Interprofessional Spine Assessment and Education Clinics (ISAEC) [8] in the province of Ontario, the Saskatchewan Spine Pathway (SSP) [9] in the provinces of Ontario, Manitoba and Saskatchewan, and the Spinal Assessment Service (SAS) in the province of Saskatchewan [10]. ISAEC is characterized by a network structure involving primary care providers (i.e., physicians and nurse practitioners), allied health providers (i.e., physiotherapists and chiropractors), and medical specialists (i.e., surgeons, pain specialists and rheumatologists) that offer evidence-based LBP assessment, education and care recommendations [8]. Only one study, a retrospective review of prospective data, investigated the effects of ISAEC clinics on LBP management. While the ISAEC model improved referral appropriateness, efficiency of magnetic resonance imaging (MRI) utilization, and wait times for surgical assessment for patients with LBP, its impact on clinical outcomes was not assessed [8]. The SSP is a model of care in which patients are referred either by a general practitioner or drawn from spine surgeons’ waiting list [9]. Specialized physiotherapists (i.e., trained in surgical triage through collaboration with spine surgeons) perform an initial assessment using a stratified care approach based on the primary pain pattern (i.e., back-dominant pain aggravated by flexion, back-dominant pain aggravated by extension, constant leg-dominant pain, and intermittent leg-dominant pain) [9]. Patients are then directed to conservative management delivered by trained primary care providers (i.e., family physicians, nurse practitioners, chiropractors and physiotherapists), to further specialized investigations or to surgical consultation [9]. Three observational studies have reported that the SSP model improves trajectory outcomes for patients with LBP, characterized by an increased tendency to use conservative approaches before being referred to a surgeon [11], reduced utilization of MRI [12] and shorter wait times for MRI when needed [11,13], as well as higher referral appropriateness for surgical care [12,13]. However, among patients who underwent surgery, pain and disability trajectories over one year were similar whether they had followed the SSP model or usual care [11]. Lastly, two observational studies and one quasi-experimental study provided evidence of the effects of the SAS on patient trajectories and clinical outcomes [10,14,15]. By introducing interface triage assessments to determine the indication of surgical consultation, this model of care achieved a surgical conversion rate (i.e., the proportion of referred patients who ultimately receive surgery) of 70.0% [10]. Patients also reported high satisfaction with the service [14], even though the intervention did not lead to significant changes in pain or disability levels four weeks after the triage assessment [15]. Nevertheless, despite these promising findings, these models of care are not widely integrated into healthcare systems, primarily due to a lack of high-quality studies supporting their effectiveness [16].

In Quebec (Canada), LBP also imposes a significant burden on the healthcare system, which operates under a physician-led delivery model with limited integration of musculoskeletal experts [17]. A retrospective analysis of medical health records revealed that 80.4% of LBP referrals for neurosurgical care at the *Centre intégré universitaire de soins de santé et de services sociaux de la Mauricie-et-du-Centre-du Québec* (CIUSSS MCQ; Quebec, Canada) were inappropriate, with 88.4% of these referrals originating from general practitioners [18]. At the same CIUSSS, over 2 500 patients were currently awaiting a neurosurgery consultation (across all conditions managed by neurosurgeons) [19], underscoring the need for an alternative model of care that leverages the skills of musculoskeletal health experts to promote timely and appropriate healthcare delivery for patients with LBP. Previous work conducted within the same clinical context has shown substantial diagnostic and management agreement between chiropractors and neurosurgeons when independently assessing patients referred for neurosurgical consultation for LBP, suggesting that chiropractors may be well positioned to contribute to triage processes [20]. However, it remains unclear whether integrating such expertise into triage pathways translates into improved patient outcomes or more efficient care trajectories.

Therefore, this study aimed to evaluate the effects of an evidence-based triage assessment performed by musculoskeletal health experts, specifically chiropractors, on clinical outcomes and care trajectories of patients with LBP referred for a neurosurgery consultation. Within the context of this study, we hypothesised that a chiropractor-informed triage would result in equivalent patient-reported clinical outcomes within the predefined equivalence margin of ±1.2 points on the NRS-11, while potentially improving care trajectories through reduced use of diagnostic tests, lower reliance on prescribed pain medication, and fewer follow-up medical visits compared with usual management.

## Methods

### Design

This study was a single-center, two-armed parallel randomised controlled trial with a 1:1 allocation ratio. The protocol (S1 Table) was registered in ClinicalTrials.gov in February 2021 (NCT04923308), and no changes to the methods were made after trial commencement. For logistical reasons related to restricted electronic medical record access, healthcare utilisation data were collected through self-reported questionnaires. This study received ethical approval from CIUSSS MCQ (CER-2021–488, 766) and the Université du Québec à Trois-Rivières (UQTR) (CER-20-269-07.13) independent research ethics boards. The trial was designed and reported in accordance with the Consolidated Standards of Reporting Trials (CONSORT) statement, including the extension for equivalence and noninferiority randomised trial designs (S2 Table) [21,22]. All study procedures were conducted in accordance with the Declaration of Helsinki [23] and the Canadian Tri-Council Policy Statement: Ethical Conduct for Research Involving Humans (TCPS 2) [24]. Written informed consent was obtained from all participants prior to their participation.

### Setting

The study was conducted in collaboration with the neurosurgery outpatient clinic affiliated to the CIUSSS MCQ. The latter serves a population of more than 530,000 people, including 82.1% over the age of 18 in 2022 [25]. Access to the neurosurgery service was managed by the Service Request Management Center (*Centre de répartition des demandes de services*-CRDS), a centralized waiting list, where patients are placed in a queue based on medical priority (defined by pre-established criteria presented in S3 Table) [26]. During the study period, the CIUSSS MCQ’s neurosurgery department was staffed by 3 neurosurgeons. Data collection was conducted at the UQTR.

### Participants

All patients scheduled at the outpatient neurosurgery clinic affiliated with the CIUSSS MCQ for LBP between July 1, 2021 and December 20, 2023 were considered potentially eligible. Patients were identified by the neurosurgery department’s administrative staff at the time of appointment scheduling based on the primary complaint listed on the referral request. Patients who provided consent were subsequently contacted by a research assistant to ascertain their eligibility and received further information about the study. Inclusion criteria were: [1] being ≥18 years of age; [2] being referred for an initial neurosurgery consultation for a primary complaint of LBP (either acute, chronic, or recurring episode); and [3] being able to legally consent to participate. Patients involved in litigation related to their LBP condition (worker’s compensation, public automobile insurance plan or other litigations) were excluded from this study.

### Interventions

Before meeting with the neurosurgeon, all participants, regardless of group allocation, underwent an evidence-based chiropractor-informed triage evaluation. Evaluations were conducted by three chiropractors employed as full-time professors or lecturers at UQTR (Quebec, Canada), each with 4–20 years of clinical experience in the management of musculoskeletal disorders. To ensure consistency in evaluation procedures and to familiarize chiropractors with the referral process to specialized care, an in-person training session was provided, including an overview of the public healthcare system’s structure, and of the CRDS’s role and pre-established criteria (see S3 Table). The triage evaluation consisted of a 2-step intervention: **[1]** Following a standardized history taking and a physical examination, participants were categorized into one of the following diagnostic groups based on their primary diagnosis (i.e., the most probable): **i)** non-specific LBP, **ii)** radicular syndrome, **iii)** specific spinal pathology, or **iv)** other causes (i.e., LBP arising from problems beyond the lumbar spine) **[2]** Participants were subsequently stratified into levels of risk of poor prognosis using the STarT Back Screening Tool (SBST), a brief prognostic screening tool that categorizes psychosocial risk for levels of pain, disability, and distress as low, medium or high [27]. Finally, the most appropriate management strategy was determined for each participant between either **i)** conservative care, **ii)** further diagnostic tests needed, **iii)** neurosurgery referral indicated, **iv)** medical emergency, and **v)** referral to another medical specialist. A detailed description of the variables considered in determining the primary diagnosis and the most suitable management strategy is provided in S4 Table.

Chiropractors only provided conclusions and recommendations to neurosurgeons. This approach was adopted because patients could only be engaged in the trial recruitment process at the time of their neurosurgical appointment, rather than at the point of referral submission, limiting the chiropractors’ opportunity to act earlier in the care trajectory. For participants in the intervention group, the triage assessment conclusions, including the primary diagnosis, risk of poor prognosis and referral recommendations, were communicated to the neurosurgeon through a clinical note (S5 Table) at the time of the patient’s consultation, without further discussion. The neurosurgeon then performed their own evaluation and determined the best management strategy. In contrast, for participants in the control group, the conclusions of the triage assessment were not shared with the neurosurgeon. Neurosurgeons performed their evaluations according to their usual clinical practice, to reflect typical real-world healthcare settings.

### Outcomes

To measure the effects of the chiropractor-informed triage, patient-reported outcomes were monitored over a one-year period (1 week, 3, 6, 12-month follow-up). In accordance with the study hypotheses, two complementary analytical frameworks were adopted.

For the equivalence hypothesis, the primary outcome was current back pain intensity, measured with the 11-point numerical rating scale (NRS-11) [28] at 3 months post-intervention. For the superiority hypothesis, secondary outcomes were used to assess whether the intervention modified patients’ clinical and healthcare trajectories over time.

Secondary outcomes were categorized as LBP clinical outcomes and patients’ trajectory outcomes. Low back pain clinical outcomes included [1] Mean back pain intensity, [2] current and mean leg pain intensity, both measured with the 11-point numerical rating scale (NRS-11) [28], ranging from 0 (no pain) to 10 (worst possible pain), [3] Low back-related disability, measured using the Oswestry Disability Index (ODI) [29,30] with scores ranging from 0 to 100, where higher scores indicate greater disability, [4] Health-related quality of life, assessed with the WHO Quality of Life-BREF (WHOQOL-BREF) [31], consisting of four domain scores ranging from 0 to 100, with higher scores indicating better quality of life [5] General expectation of recovery, measured using the NRS-11 by asking patients: “*How likely do you think it is that you will have a complete recovery*” with possible answers ranging from −5 (very unlikely) to +5 (very likely), with 0 being “ *I don’t know*”, [6] Perceived global rating of change, measured using the 7-point scale Patient Global Impression of change [32,33], ranging from 1 (very much worse) to 7 (very much improved) and [7] Patient satisfaction with the triage assessment, measured at baseline only using items 5–9 from the Visit-Specific Satisfaction Instrument (VSQ9) [34], transformed to a 0–100 scale, with higher scores indicating greater satisfaction. Patients’ trajectory outcomes included [1] the diagnosis and clinical management established by the neurosurgeon, retrieved from the patient’s medical file after the neurosurgery consultation (categorical variables), [2] Patient-reported use of medical services for their current LBP episode since the previous follow-up (binary variables), including i) imaging exams, ii) laboratory tests, and iii) medical prescription (participants indicated whether or not they had undergone each type of service since the last assessment), [3] consultation with a general practitioner (GP) (patients were asked at follow-ups whether they sought the expertise of a GP for their current LBP episode since the last assessment time point), and [4] use of non-publicly funded care (patients were asked at follow-ups whether they sought non-publicly funded care for their current LBP episode since the last assessment time point). Sociodemographic data were also collected at baseline via a standardized questionnaire, including demographic characteristics (age, sex, gender, Indigenous identity, ethnic minority status, and religion), social characteristics (marital status and employment status), and socioeconomic indicators (educational attainment, annual income, and private insurance coverage).

### Harms

Adverse events were not assessed systematically. Nevertheless, participants were instructed to contact the research team if they had any questions or concerns regarding their condition.

### Sample size

The sample size calculation was based on an equivalence study design, intended to demonstrate that the new approach (chiropractor-informed group) led to equivalent clinical outcomes compared to usual care (not chiropractor-informed group). The primary outcome was back pain intensity on a 0–10 numeric rating scale at 3 months. Equivalence was defined as the two-sided 95% confidence interval for the between-group mean difference (chiropractor-informed minus usual care) lying entirely within −1.2 to +1.2 points (equivalence margin Δ = 1.2). This margin was deliberately set narrower than the commonly accepted minimal clinically important difference (MCID) of around 2 points [35], providing a conservative threshold to reduce the risk of incorrectly concluding equivalence when a meaningful difference may exist. Assuming a common SD of 2.0 points [36] and a true mean difference of 0, a 1:1 allocation with the two one-sided tests (TOST) procedure (α = 0.025 per one-sided test) required n = 80 participants per group to achieve 80% power. Allowing 15% attrition, we planned to recruit 70 participants per group (N = 140).

### Randomisation and blinding

Randomisation determined whether the neurosurgeon received the triage assessment conclusions following the triage process. Block randomisation with blocks of 10 was used to allocate participants to the study groups. The randomisation sequence was computer-generated, and group assignments were concealed in sequentially numbered sealed opaque envelopes, managed by a research assistant who was blinded to the study objectives. Both chiropractors conducting the triage evaluations and the patients were blinded to group allocation. Chiropractors remained unaware of the participant’s assignment until completing their assessment.

### Statistical analysis

The distribution of continuous variables was assessed using the Shapiro-Wilk test and visual inspection of histograms and Q-Q plots to evaluate departures from normality. As continuous variables met the assumptions of normality, parametric tests were used for all comparisons involving continuous outcomes. Between-group comparisons at baseline were performed using independent-samples t tests for continuous variables and chi-square tests for categorical variables. For the equivalence hypothesis, between-group comparisons of pain outcomes at the primary endpoint (3 months) were conducted using independent-samples *t* tests on pain change scores. Equivalence was evaluated using the TOST procedure, with equivalence margins set at ±1.2 points on the NRS-11. Equivalence was concluded when the 95% confidence interval of the difference in pain change scores (Δ intervention − Δ control) was entirely contained within the predefined equivalence bounds. Missing follow-up data were handled using a multiple imputation approach based on random forest imputation, implemented through the miceRanger package in R (version 4.5.1) [37]. This method combines the flexibility of multivariate imputation by chained equations (MICE) with the predictive performance and robustness of tree–based ensemble models [37]. The imputed datasets were combined into a single analytical dataset, on which the statistical analyses were performed. Repeated-measures ANOVAs were conducted with group (intervention vs control) as the between-subject factor and time as the within-subject factor to evaluate changes in clinical outcomes over time. The primary effect of interest was the group × time interaction, which assessed whether changes over time differed between groups. Bonferroni-adjusted post hoc tests were performed when significant group × time effects were observed to further explore between-group differences at specific time points. For these analyses, a two-tailed p value < 0.05 indicated statistical significance, while the MCID was used to determine whether observed changes were clinically meaningful. In addition, generalized linear mixed models with a binomial distribution were used to compare changes in care trajectories outcomes over time between groups. Models included group, time, and the group × time interaction as fixed effects, with participant included as a random effect to account for repeated measurements. The group × time interaction was used to evaluate whether changes in care trajectories over time differed between groups. All statistical analyses were performed using IBM SPSS Statistics for Windows, version 18.0.0 (Armonk, NY: IBM Corp.) and followed the intention-to-treat principle, with participants analyzed according to randomly assigned group. Given the absence of protocol deviations or crossovers, no separate per-protocol analyses were conducted.

## Results

### Recruitment

**Fig 1:**
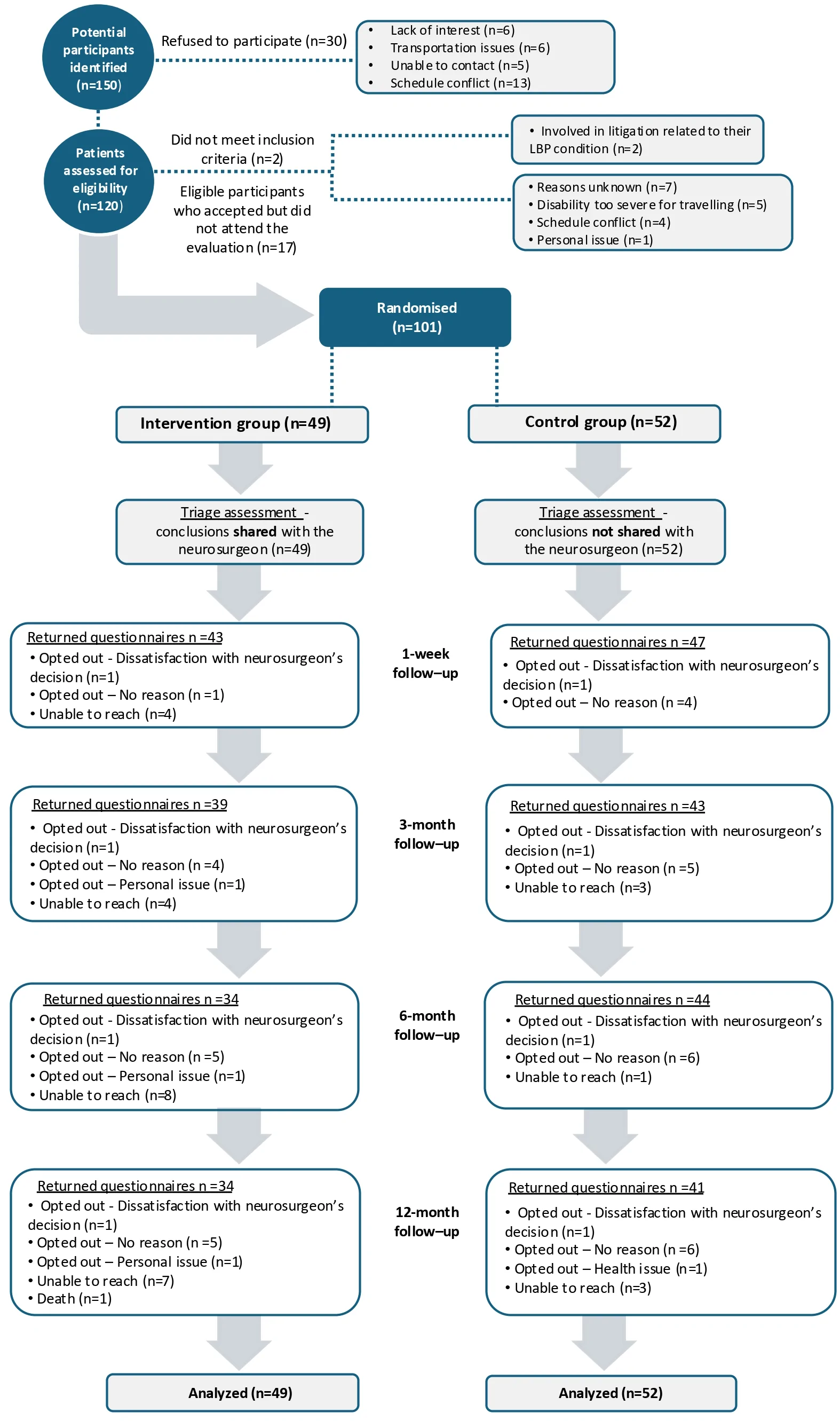
Flowchart of study participants. Flow of participants from enrollment to final analysis.

Between July 1, 2021, and December 20, 2023, 150 eligible patients were contacted, of whom 118 (78.7%) agreed to participate. Of these, 17 patients did not attend the triage assessment, leading to 101 participants that were randomly assigned to the intervention group (n = 49) or the control group (n = 52). Fig 1 presents, for each group, the flow of participants including the numbers and reasons for attrition and non-participation. Recruitment was ultimately stopped due to the challenges of scheduling assessments on short notice before neurosurgery appointments and because preliminary analyses suggested that the addition of participants was unlikely to lead to meaningful differences between groups.

### Participants ‘characteristics

Table 1 and 2 present baseline demographic and clinical characteristics for each group. No significant difference between groups were found in baseline characteristics.

**Table 1:** Baseline characteristics.

	Intervention group (n = 49)	Control group (n = 52)	*p-value*
Sociodemographic data n (%) or Mean ± SD			
Age (years)	61.8 ± 13.4	58.9 ± 15.8	0.3
Sex *Male* *Female*	25 (51.0) 24 (46.2)	28 (53.8) 24 (49.0)	0.8
Gender, n (%) *Male* *Female* *Other*	23 (46.9) 24 (49.0) 2 (4.1)	26 (50.0) 23 (44.2) 3 (5.8)	0.9
Member of an indigenous community (*yes)*	2 (4.1)	5 (9.6)	0.3
Belonging to an ethnic minority group (*yes*)	0 (0)	0 (0)	–
Religion *Christianism* *Atheism* *Other*	39 (79.6) 7 (14.3) 3 (6.1)	41 (80.4) 8 (15.7) 2 (3.8)	0.7
Marital status *Married* *In a relationship – not married* *Single* *Divorced* *Widowed*	30 (61.2) 5 (10.2) 7 (14.3) 5 (10.2) 2 (4.1)	27 (51.9) 8 (15.4) 7 (13.5) 5 (9.6) 5 (9.6)	0.7
Employment status *Full time* *Part time* *Retired* *At home* *Temporary sick leave* *Welfare beneficiary*	16 (33.3) 5 (10.4) 21 (42.9) 2 (4.2) 4 (8.3) 0 (0.0)	11 (21.2) 1 (1.9) 25 (48.1) 1 (1.9) 11 (21.2) 3 (5.8)	0.07
Highest educational attainment *Primary (elementary) school education* *Secondary education (high school)* *College degree (pre-university or technical diploma)* *Trade school (Technical or occupational certificate)* *Undergraduate degree (Bachelor or advanced diploma)* *Graduate degree* *Other*	9 (18.8) 13 (27.1) 7 (14.6) 13 (27.1) 4 (8.3) 0 (0.0) 2 (4.2)	4 (7.8) 18 (35.3) 13 (25.5) 8 (15.7) 6 (11.8) 1 (2.0) 1 (2.0)	0.3
Annual personal income ($ CAD) < 10 000 10 000-29 999 30 000- 49 999 50 000- 69 999 70 000- 89 999 90 000-109 999 110 000-129 999 ≥ 130 000 Prefers not to answer	0 (0.0) 16 (32.7) 12 (24.5) 3 (6.1) 3 (6.1) 1 (2.0) 3 (6.1) 1 (2.0) 10 (20.4)	3 (5.9) 18 (35.3) 15 (29.4) 6 (11.8) 2 (3.8) 2 (3.8) 2 (3.8) 2 (3.8) 1 (2.0)	0.1
Private insurance coverage *Prescription drugs* *Imaging* *Privately funded healthcare*	27 (55.1) 21 (41.2) 12 (24.5)	24 (47.1) 11 (22.4) 17 (33.3)	0.4 0.07 0.6

CAD: Canadian dollar; LBP: Low back pain; SD: Standard deviation.

**Table 2:** Clinical profiles at baseline.

	Intervention group (n = 49)	Control group (n = 52)	*p-value*
Primary outcomes	Mean ± SD	Mean ± SD	*p*
Current back pain intensity (/10)	5.3 ± 2.6	5.2 ± 2.5	0.8
Secondary outcomes *– LBP clinical outcomes*	**n (%) or Mean** ± **SD**	**n (%) or Mean** ± **SD**	***p***
Mean back pain intensity (/10)	6.1 ± 2.3	6.4 ± 2.7	0.5
Current leg pain intensity (/10)	4.2 ± 2.8	4.3 ± 2.9	1.0
Mean leg pain intensity (/10)	5.5 ± 2.6	5.5 ± 2.8	1.0
Low back-related disability (/100)	41.1 ± 16.9	37.7 ± 17.1	0.3
Health-related QoL (/100) *Physical domain* *Psychological domain* *Social domain* *Environmental domain*	45.4 ± 20.3 61.2 ± 16.8 64.9 ± 15.8 72.0 ± 13.8	44.0 ± 20.7 63.8 ± 16.3 67.1 ± 19.2 74.4 ± 16.1	0.7 0.5 0.6 0.4
General expectation of recovery, n (%) *−5 (very unlikely)* *−4* *−3* *−2* *−1* *0 (I don’t know)* *1* *2* *3* *4* *5 (very likely)*	15 (31.3) 4 (8.3) 4 (8.3) 2 (4.2) 1 (2.1) 12 (25.0) 2 (4.2) 4 (8.3) 3 (6.3) 0 (0.0) 1 (2.1)	9 (17.6) 2 (3.9) 7 (13.7) 3 (5.9) 1 (2.0) 18 (35.3) 2 (3.9) 1 (2.0) 2 (3.9) 2 (3.9) 4 (7.8)	0.4
Risk of poor prognosis (SBST) *Low risk* *Medium risk* *High risk*	11 (22.4) 23 (46.9) 15 (30.6)	14 (26.9) 20 (38.5) 18 (34.6)	0.7
Patient satisfaction (/100)	97.3 ± 5.7	99.1 ± 2.8	0.05
Secondary outcomes – *Patients’ trajectory outcomes*	**n (%)**	**n (%)**	***p***
Neurosurgeon’s diagnosis, n (%) *Non-specific LBP* *Radicular syndrome* *Specific LBP* *Other*	21 (45.7) 25 (54.3) 0 (0) 4 (8.2)	21 (40.4) 27 (51.9) 2 (3.8) 2 (3.8)	0.4
Neurosurgeon’s management strategy, n (%) *Conservative care* *Further diagnostic tests needed* *Neurosurgery referral indicated* *Falls under another specialty*	25 (52.1) 1 (2.1) 21 (43.8) 1 (2.1)	26 (50.0) 2 (3.8) 21 (40.4) 3 (5.8)	0.8

LBP: Low back pain; QoL: Quality of life; SD: Standard deviation; SBST: STarT Back Screening Tool.

### Equivalence testing of primary pain outcomes

At the primary endpoint (3 months), the mean change from baseline in current back pain intensity was −0.47 ± 2.44 in the control group and −0.03 ± 2.56 in the intervention group. The mean difference in change scores between groups (Δ intervention − Δ control) was 0.44 ± 0.54 (95% CI −1.51-0.63) Based on the predefined equivalence framework using the two one-sided tests (TOST) procedure and an equivalence margin of ±1.2 points on the NRS-11, equivalence between groups could not be concluded for current back pain intensity at 3 months as the confidence interval extended beyond the equivalence bounds.

### Intervention effect on clinical outcomes

#### Back pain intensity.

Current and mean back pain intensity scores decreased significantly over time in both groups (p < 0.05). Current back pain intensity scores at 12 months were significantly lower than both baseline (–0.9 ± 0.3; p = 0.01) and 1-week follow-up (–0.8 ± 0.2; p = 0.006). Overall, the control group showed greater improvements than the intervention group (F (1, 99) = 4.25, p = 0.04, η² = 0.04). A significant group × time interaction (F (3.42, 339) = 3.08, p = 0.02, η² = 0.03) was observed, reflecting larger decreases in current back pain intensity in the control group at both 6-month (mean difference: 1.3 ± 0.5; p = 0.02) and 12-month (mean difference: 1.6 ± 0.6; p = 0.003) follow-ups. For mean back pain intensity, no main effect of group or group x time interaction was found (p ≥ 0.05). Detailed results are shown in Table 3. Changes in both current and mean back pain intensity are not considered clinically meaningful, as a minimum 2-point difference on the NRS-11 is generally required to reach MCID [35].

**Table 3:** Intervention effects on back pain intensity.

Outcome	Timepoint	Control group (C)	Intervention group (I)	Mean difference	95% CI	*p*
		Mean ± SD (95% CI)		(I – C) ± SD		
Current back pain intensity	**Group effect**	4.4 ± 0.3 (3.8-5.0)	5.3 ± 0.3 (4.7-5.9)	0.9 ± 0.4	0.08 −1.7	0.03*
	Baseline	5.2 ± 0.4 (4.5-5.9)	5.3 ± 0.4 (4.6-6.0)	0.1 ± 0.5	−0.9–1.1	0.8
	1-week	4.6 ± 0.3 (4.0-5.3)	5.5 ± 0.3 (4.9-6.2)	0.9 ± 0.5	−0.02–1.8	0.05
	3-month	4.6 ± 0.4 (3.9-5.4)	5.3 ± 0.4 (4.5-6.1)	0.7 ± 0.5	−0.4–1.7	0.2
	6-month	4.0 ± 0.4 (3.3-4.8)	5.3 ± 0.4 (4.5-6.1)	1.3 ± 0.5	0.2–2.3	0.02*
	12-month	3.5 ± 0.4 (2.8-4.2)	5.1 ± 0.4 (4.4-5.9)	1.6 ± 0.5	0.6–2.7	0.003*
Mean back pain intensity	**Group effect**	5.1 ± 0.3 (4.6-5.7)	5.7 ± 0.3 (5.1-6.3)	0.6 ± 0.4	−1.4–0.2	0.2
	Baseline	6.4 ± 0.3 (5.7-7.1)	6.1 ± 0.4 (5.4-6.8)			
	1-week	5.3 ± 0.3 (4.6-5.9)	6.0 ± 0.3 (5.4-6.7)			
	3-month	5.2 ± 0.3 (4.6-5.9)	5.7 ± 0.3 (5.0-6.4)			
	6-month	4.6 ± 0.4 (3.8-5.3)	5.6 ± 0.4 (4.8-6.4)			
	12-month	4.2 ± 0.4 (3.5-5.0)	5.3 ± 0.4 (4.5-6.1)			

SD: Standard deviation; * p < 0.05.

### Leg pain intensity

For both current and mean leg pain intensity, no main effect of group or group × time interaction was found (p ≥ 0.05). A significant effect of time was found only for leg pain reported over the past week (F (3.52, 352) = 7.82, p < 0.001, η² = 0.07), with significant reductions observed from baseline to 6 months (–1.0 ± 0.3; p = 0.002) and from baseline to 12 months (–1.4 ± 0.3; p < 0.001). However, these differences are not clinically meaningful. Table 4 presents within-group descriptive data across time.

**Table 4:** Intervention effects on leg pain intensity.

Outcome	Timepoint	Control group (C)	Intervention group (I)	Mean difference	95% CI	*p*
**Mean ± SD (95% CI)**		**(I – C) ± SD**
Current leg pain intensity	**Group effect**	3.8 ± 0.3 (3.1-4.4)	4.6 ± 0.3 (3.9-5.3)	0.8 ± 0.5	−0.1–1.7	0.08
	Baseline	4.2 ± 0.4 (3.4-5.0)	4.2 ± 0.4 (3.4-5.0)			
	1-week	3.9 ± 0.4 (3.2-4.7)	5.2 ± 0.4 (4.5-6.0)			
	3-month	4.2 ± 0.4 (3.4-5.0)	4.5 ± 0.4 (3.6-5.3)			
	6-month	3.5 ± 0.4 (2.7-4.3)	4.5 ± 0.4 (3.6-5.3)			
	12-month	3.1 ± 0.4 (2.2-4.0)	4.6 ± 0.5 (3.7-5.5)			
Mean leg pain intensity	**Group effect**	5.1 ± 0.3 (4.6-5.7)	5.7 ± 0.3 (5.1-6.3)	0.6 ± 0.4	−1.4–0.2	0.2
	Baseline	6.4 ± 0.3 (5.7-7.1)	6.1 ± 0.4 (5.4-6.8)			
	1-week	5.3 ± 0.3 (4.6-5.9)	6.0 ± 0.3 (5.4-6.7)			
	3-month	5.2 ± 0.3 (4.6-5.9)	5.7 ± 0.3 (5.0-6.4)			
	6-month	4.6 ± 0.4 (3.8-5.3)	5.6 ± 0.4 (4.8-6.4)			
	12-month	4.2 ± 0.4 (3.5-5.0)	5.3 ± 0.4 (4.5-6.1)			

SD: Standard deviation; * p < 0.05.

### Low back-related disability

No main effect of group (p = 0.1) or group × time interaction (p = 0.06) was found for low back-related disability. A significant time effect was observed (F (2.91, 290) = 10.38, p < 0.001, η² = 0.10), indicating progressive improvement across all follow-up assessments, with a mean difference of 8.85 ± 1.86 points at 12 months compared to baseline (*p* < 0.001). However, such difference is not considered clinically meaningful, as a minimum 20-point difference on the ODI is required to reach the minimal clinically important difference (MCID) [35]. Table 5 presents within-group descriptive data across time.

**Table 5:** Intervention effects on low back-related disability.

Timepoint	Control group (C)	Intervention group (I)	Mean difference	95% CI	*p*
	Mean ± SD (95% CI)		(I – C) ± SD		
Group effect	32.4 ± 2.4 (27.6-37.1)	37.8 ± 2.5 (32.9-42.6)	5.4 ± 3.4	−1.4–12.2	0.1
Baseline	37.7 ± 2.3 (33.0-42.3)	41.1 ± 2.4 (36.3-45.9)			
1-week	33.7 ± 2.4 (28.9-38.5)	38.8 ± 2.5 (33.8-43.7)			
3-month	33.5 ± 2.6 (28.3-38.7)	35.8 ± 2.7 (30.5-41.2)			
6-month	31.6 ± 2.9 (25.9-37.4)	37.0 ± 3.0 (31.1-43.0)			
12-month	25.4 ± 3.1 (19.3-31.5)	36.1 ± 3.2 (29.8-42.4)			

SD: Standard deviation; * p < 0.05.

### Quality of life

There was no main effect of group or group × time interactions for any of the quality-of-life outcomes. Both groups showed significant improvements over time in the physical quality of life domain (F (2.92, 292) = 13.47, p < 0.001, η² = 0.12), with increases in WHOQOL-Bref scores from baseline to 6 months (+6.84 ± 2.09; p = 0.02) and from baseline to 12 months (+10.39 ± 2.18; p < 0.001). Significant time effects were also observed for the psychological (F (3.27, 327) = 3.46, p = 0.01, η² = 0.03) and social (F (3.27, 327) = 5.76, p < 0.001, η² = 0.06) quality of life domains. Both domains displayed variability over time, with alternating improvements and declines. Social scores were significantly lower at 1 week (−8.49 ± 2.13; p = 0.001) and 6 months (−5.21 ± 1.73; p = 0.03) compared with baseline measures. By 12 months, the psychological domain showed a slight, non-significant improvement from baseline, whereas the social domain remained below baseline levels. No significant effects were observed for the environmental domain. Table 6 presents within-group descriptive data for each quality-of-life domains across time.

**Table 6:** Intervention effect on quality-of-life domains.

Outcome	Timepoint	Control group (C)	Intervention group (I)	Mean difference	95% CI	*p*
		Mean ± SD (95% CI)		(I – C) ± SD		
Physical domain (/100)	**Group effect**	50.5 ± 2.5 (45.5-55.5)	47.3 ± 2.6 (42.2-52.5)	−3.2 ± 0.4	−10.2–4.0	0.4
	Baseline	44.1 ± 2.8 (38.4-49.7)	45.6 ± 2.9 (39.8-51.4)			
	1-week	45.9 ± 2.8 (40.5-51.4)	43.4 ± 2.8 (37.8-49.1)			
	3-month	49.8 ± 2.8 (44.3-55.3)	47.0 ± 2.9 (41.3-52.6)			
	6-month	53.3 ± 3.2 (46.9-59.6)	49.5 ± 3.3 (43.0-56.1)			
	12-month	59.4 ± 3.2 (53.1-65.8)	51.1 ± 3.3 (44.6-57.7)			
Psychologi-cal domain (/100)	**Group effect**	62.9 ± 2.3 (58.4-67.4)	59.5 ± 2.3 (54.8-64.1)	−3.4 ± 3.3	−9.9–3.1	0.3
	Baseline	63.6 ± 2.3 (59.1-68.1)	61.2 ± 2.3 (56.6-65.9)			
	1-week	59.1 ± 2.8 (53.6-64.6)	57.3 ± 2.9 (51.6-63.0)			
	3-month	64.2 ± 2.6 (59.1-69.3)	59.9 ± 2.6 (54.6-65.1)			
	6-month	61.9 ± 2.8 (56.2-67.5)	57.9 ± 2.9 (52.1-63.7)			
	12-month	65.7 ± 2.7 (60.3-71.1)	61.1 ± 2.8 (55.6-66.7)			
Social domain (/100)	**Group effect**	62.9 ± 2.2 (58.6-67.2)	59.9 ± 2.2 (55.5-64.3)	−3.0 ± 3.1	−9.1–3.2	0.3
	Baseline	66.9 ± 2.4 (62.1-71.7)	64.9 ± 2.5 (59.9-70.0)			
	1-week	58.8 ± 3.0 (52.9-64.7)	56.0 ± 3.1 (49.9-62.0)			
	3-month	61.9 ± 2.5 (57.0-66.8)	59.9 ± 2.6 (54.8-65.0)			
	6-month	62.5 ± 2.8 (57.0-68.0)	58.8 ± 2.9 (53.1-64.4)			
	12-month	64.3 ± 2.7 (58.9-69.7)	60.1 ± 2.8 (54.6-65.7)			
Environment-al domain (/100)	**Group effect**	73.5 ± 2.0 (69.6-77.4)	70.0 ± 2.0 (66.0-74.1)	−3.5 ± 2.8	−9.1–2.2	0.2
	Baseline	74.3 ± 2.1 (70.2-78.4)	72.0 ± 2.1 (67.8-76.2)			
	1-week	72.0 ± 2.3 (67.4-76.6)	69.4 ± 2.4 (64.7-74.1)			
	3-month	72.7 ± 2.2 (68.3-77.1)	70.9 ± 2.3 (66.3-75.4)			
	6-month	72.8 ± 2.4 (68.0-77.6)	69.0 ± 2.5 (64.1-74.0)			
	12-month	75.7 ± 2.6 (70.5-80.8)	69.0 ± 2.7 (63.7-74.2)			

SD: Standard deviation; * p < 0.05.

### Perception of change

Table 7 presents the distribution of participants’ perceived change (improved, unchanged, or worsened) in the intervention and control groups. At 1, 3, 6, and 12 months, both groups showed similar proportions reporting improvement, no change, or deterioration, with no statistically significant between-group differences at any time point (all p ≥ 0.05). Overall, the proportion of participants reporting improvement increased from 1 week to 3 months in the intervention (from 14.3% to 40.8%) and control (from 19.2% to 38.5%) groups, then stabilized or fluctuated slightly through 6–12 months.

**Table 7:** Participants’ perceived global change over time by group.

Global impression of change	Intervention group (n = 49)	Control group (n = 52)	*p*
1-week follow-up (n, %)			
Improved	7 (14.3)	10 (19.2)	0.4
Unchanged	33 (67.3)	37 (71.2)	
Worsened	9 (18.4)	5 (9.6)	
3-month follow-up (n, %)			
Improved	20 (40.8)	20 (38.5)	0.6
Unchanged	19 (38.8.)	17 (32.7)	
Worsened	10 (20.4)	15 (28.8)	
6-month follow-up (n, %)			
Improved	16 (32.7)	16 (30.8)	0.7
Unchanged	20 (40.8)	18 (34.6)	
Worsened	13 (26.5)	18 (34.6)	
12-month follow-up (n, %)			
Improved	17 (34.7)	20 (38.5)	0.6
Unchanged	17 (34.7)	21 (40.2)	
Worsened	15 (30.6)	11 (21.2)	

Counts and column percentages of participants reporting improvement, no change, or deterioration at each follow-up.

### Intervention effect on healthcare trajectory outcomes

**Fig 2:**
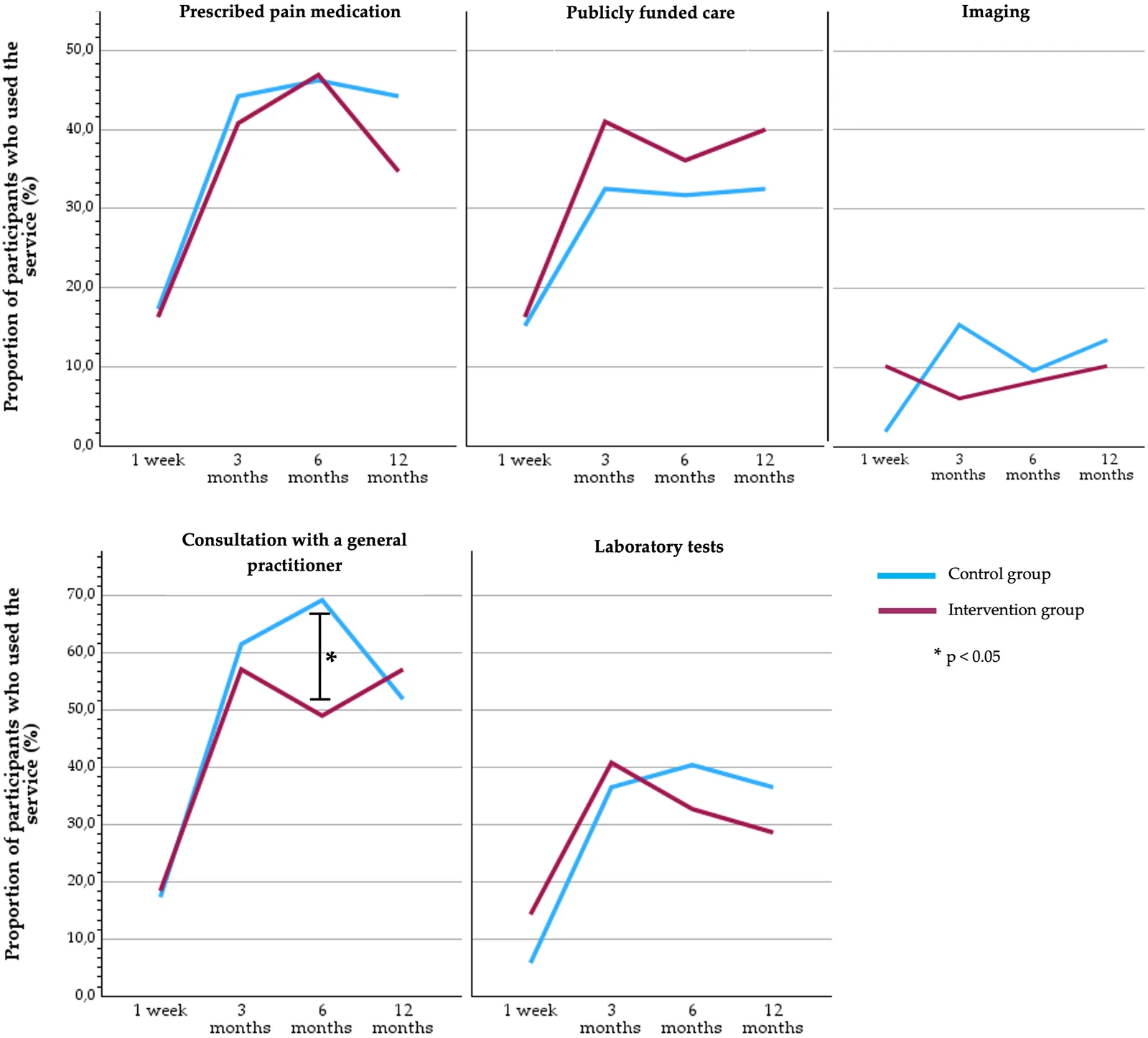
Healthcare service utilization over time. Proportion of participants reporting use of healthcare services over time in the intervention and control groups. An asterisk (*) indicates a statistically significant between-group difference at the corresponding time point (*p* < 0.05).

Fig 2 presents the proportion of participants in each group who reported using healthcare resources since the previous assessment. No significant between-group differences were observed for the use of publicly funded care, prescribed pain medication, or imaging and laboratory investigations. At the 6-month follow-up, a significantly smaller proportion of participants in the intervention group (49.0%) reported consulting a general practitioner since the 3-month follow-up compared with 69.2% of participants in the control group (p = 0.04). However, this difference was not maintained at 12 months, and generalized linear mixed models revealed no significant group effects or group × time interactions, suggesting that both groups followed similar care trajectories over time. Although the differences were not statistically significant, participants in the control group tended to have slightly lower odds of using non–publicly funded care at the 3-month follow-up, and slightly higher odds of having consulted a general practitioner between the 3- and 6-month assessments. Significant time effects were observed for the use of non–publicly funded care (p = 0.03), prescribed pain medication (p < 0.001), laboratory tests (p < 0.001), and for consultations with a general practitioner (p < 0.001), reflecting that the odds of using healthcare resources varied significantly across time points in both groups. Specifically, the overall odds of consulting with a general practitioner (OR 5.92; 95% CI [2.56–13.70], p < 0.001) were significantly higher 12 months after the triage evaluation compared to 1-week follow-up.

### Harms

No adverse events or unintended effects were observed during the triage evaluation nor were any reported during the week following the assessment.

## Discussion

This study aimed to evaluate the effects of an evidence-based triage assessment performed by chiropractors on the clinical outcomes and care trajectories of patients with LBP referred for a neurosurgery consultation. It was hypothesized that this intervention would lead to equivalent clinical outcomes while optimizing care trajectories compared to usual management.

Equivalence between groups for back pain outcomes could not be demonstrated based on the predefined margins. However, the observed differences at each time point remained below the minimal clinically important difference, suggesting clinical similarity between groups. Overall, both groups experienced small but statistically significant improvements over time across most clinical outcomes, with no consistent evidence that the triage intervention led to superior clinical benefits compared with usual care. These results were expected given that chiropractors were responsible for providing conclusions and recommendations to neurosurgeons, a role intended to guide rather than directly change clinical decisions.

The triage assessment appeared to have a modest, short-term influence on care trajectories, with participants in the intervention group reporting lower rates of consultation with a general practitioner at the 6-month follow-up (p = 0.04). However, the mixed model analysis accounting for repeated measures and intra-subject correlation showed no statistically significant differences, suggesting that this effect was not sustained over time. The lower proportion of patients reporting general practitioner consultations in the intervention group may reflect the influence of the shared triage recommendations. Evidence from a systematic review including 11 prospective cohort studies suggests that recovery from a new episode of LBP typically occurs within the initial 6–12 weeks, after which persistent symptoms are less likely to resolve spontaneously [38]. Therefore, the 6-month difference may represent a stage at which persistent symptoms in the control group prompted greater reliance on medical consultations. Providing neurosurgeons with the chiropractor’s assessment and management conclusions may have promoted evidence-based care, and participants in the intervention group may have been more likely to seek conservative care approaches for new episodes of LBP.

To optimize the potential benefits of the triage assessment on clinical outcomes and care trajectories, evidence suggests that priority should be given to minimizing inappropriate referrals and promoting timely access to the most appropriate healthcare professional [39]. This perspective is particularly relevant when interpreted alongside our study results. Indeed, a secondary analysis of our study findings showed that neurosurgeons recommended conservative care as the most appropriate management strategy for 51.0% of participants [20]. Among these patients, nearly 70% had been waiting for more than six months to see the neurosurgeon, and almost one quarter for over twelve months [20]. Informally, several of these patients reported having neither initiated conservative treatment nor engaged in self-management strategies during the waiting period, mentioning uncertainty about appropriate interventions and concern about exacerbating their condition. Furthermore, among patients considered surgical candidates, about one third were seen after seven months or more [20]. These results not only suggest that non-surgical patients are denied timely and appropriate care for their condition, but also that suboptimal referral practices contributed to long and avoidable delays for those in need of spine surgery. Collectively, these results suggest that an evidence-based triage service led by musculoskeletal experts might have a greater impact on clinical outcomes and care trajectories if implemented earlier in the care pathway, either within primary care settings or as an intermediate assessment service.

Interestingly, most studies assessing the effectiveness of LBP clinical pathways with intermediate services have largely focused on system-level outcomes, such as patient flow and healthcare resource utilization, while evidence on clinical outcomes remains limited [40]. Reported improvements in care trajectories included greater appropriateness of referrals to spinal surgeons [8,10,12,13,15,41–44], higher use of conservative treatments prior to surgical consultation [11], reduced use of unnecessary imaging [12,13,45], similar or shorter wait times to access secondary or tertiary care services [12,13,43,46,47], and fewer subsequent consultations with general practitioners [48]. To date, only one randomised controlled trial investigated the effects of a stratified care pathway, implemented as an interface service, on the resolution of LBP and sciatica symptoms [46]. Compared with patients receiving usual care, patients managed through the stratified care model showed no significant differences in median time to symptoms resolution, nor in back or leg pain intensity, physical function, anxiety, depression, and kinesiophobia symptoms at both 4-month and 12-month follow-ups [46]. Another prospective non-randomised study compared the clinical profiles and lumbar spine surgery outcomes of patients referred to a spine surgeon through a multidisciplinary spine care pathway (SSP) with those who accessed the service by conventional referral processes [11]. The SSP assessment did not result in significant differences in LBP intensity or functional status of patients at initial presentation to the spine surgeon, nor did it lead to different surgical outcomes [11]. However, patients referred through the SSP tended to experience shorter waiting times for both the surgical consultation and subsequent surgery [11]. Finally, an observational study by [Jess et al. (2021)] reported that patients with LBP of less than 3 months duration enrolled in the North East of England Regional Back Pain and Radicular Pain Pathway demonstrated larger clinical improvements in pain intensity, disability and quality of life at 12-month follow-up compared with patients enrolled in the pathway but experiencing symptoms for 12 months or longer [49]. Although this study did not include a usual care comparator group, these findings suggest that triage services may achieve greater effectiveness when introduced early in the patient management process, potentially preventing the transition to chronicity. Nonetheless, additional research is warranted to determine the clinical effectiveness of triage services led by musculoskeletal experts prior to advocating their implementation in primary care or as intermediate interface models.

Future research should also examine potential barriers to implementing alternative care models to ensure their clinical applicability and acceptability. Some authors have reported resistance from healthcare professionals who either bypassed established care pathways or expressed reluctance toward collaborative models, perceived as diminishing their autonomy or competencies [10,41]. Additionally, clinicians have reported frustration over their limited ability to influence the timing of subsequent treatments once patients entered the alternative pathway [50]. Some patients also perceived these models as overly protocol-driven, reducing their participation in shared decision-making [51], or described their experience as fragmented, occasionally resulting in conflicting opinions or additional delays to access services [51]. Overall, these observations underscore that the successful implementation of triage services requires the active engagement of all stakeholders, including administrators, clinicians, and patients, to promote collaboration, shared decision-making, and a coordinated care experience.

### Strengths and limitations

This study advances understanding of the effects of an evidence-based triage intervention on clinical outcomes and care trajectories for patients referred to tertiary care for LBP. Given that prior research on the integration of musculoskeletal experts into care pathways has mostly relied on observational studies, the present work enhances the quality and robustness of available data. However, some limitations should be acknowledged. Although standardized assessment methods were applied to minimize variability among chiropractors, inter-rater reliability was not directly measured. Beyond this methodological consideration, a qualitative assessment involving neurosurgeons could have provided valuable insights into whether the reports were perceived as useful and whether they influenced clinical decision-making. The external validity of the study is also limited by its single-site setting, the small number of participating chiropractors and neurosurgeons, and the relatively homogeneous sociodemographic profile of participants. As a result, the findings may not fully represent the broader population of patients referred for specialized low back pain management. Furthermore, although the chiropractor’s conclusions and recommendations were withheld from neurosurgeons in the control group, contamination cannot be ruled out, as participants may have shared information from their assessment during the consultation, potentially reducing between-group differences. Lastly, care trajectories data were based on self-reported measures and may therefore be subject to recall bias. Additionally, these data were not analyzed according to patients’ psychosocial risk profiles or diagnostic categories, preventing assessment of how care patterns differed across patient subgroups.

## Conclusion

This study examined the impact of a novel, evidence-based triage intervention led by chiropractors on clinical outcomes and care trajectories for patients referred to tertiary care for LBP. Despite not meeting formal equivalence criteria, the consistently small differences observed between groups over time suggest that the triage intervention performs similarly to usual management. Although this may indicate that the chiropractors’ involvement did not substantially alter patient trajectories, evidence from previous work suggest that these findings may instead reflect the high level of concordance between chiropractors’ and neurosurgeon’s clinical decisions. In this context, future studies should explore the implementation of triage services led by musculoskeletal experts earlier in the care pathway, either within primary care or as an intermediate assessment service, where their potential to optimize patient trajectories may be greater.

## Supporting information

S1 Table Grant Application. Study protocol.
(PDF)

S2 Table CONSORT 2025 checklist. Checklist item descriptions and corresponding page numbers.
(PDF)

S3 Table Adult Neurosurgery Consultation. Summary of the referral criteria used by the CRDS to determine eligibility and prioritization for neurosurgical consultation.
(PDF)

S4 Table Variables used to inform diagnostic and management strategies. Variables considered by chiropractors in determining the primary diagnosis and best management strategy.
(DOCX)

S5 Table Clinical Note. Clinical note used to communicate the chiropractors’ assessment findings and recommendations to the neurosurgeons for participants in the intervention group.
(PDF)
