# Supplementary material for: Effects of musculoskeletal expert input on low back pain outcomes: A randomised controlled trial
Source: PLoS One. 2026 Aug 26;21(8):e0356604. doi: 10.1371/journal.pone.0356604 (PMC13513973; doi:10.1371/journal.pone.0356604)
Supplement: Supplementary file 1 [file pone.0356604.s001.pdf]

Grant Application

Principal Investigator:

**Surname:**	Descarreaux
**Given Name(s)**	Martin
**Position:**	Full professor
**Appointment Held:**	Full professor, Department of Human kinetics, UQTR
**E-mail:**	martin.descarreaux@uqtr.ca
**Telephone #:**	(819) 376-5011 ext: 3791

Co-Principal Investigator:

**Surname:**	Marchand
**Given Name(s)**	Andrée-Anne
**Position:**	Professor (non-tenure track)
**Appointment Held:**	Professor, Department of Chiropractic, UQTR
**E-mail:**	andree-anne.marchand@uqtr.ca
**Telephone #:**	(819) 376-5011 ext: 4482

Co-Investigator(s):

**Name:**	Cesar Hincapié	**Position:**	Senior scientist, University of Zurich
**Name:**	Petra Schweinhardt	**Position:**	Head of research, University of Zurich
**Name:**	Marie Beauséjour	**Position:**	Associate professor, Université de Sherbrooke
**Name:**	Claude-Édouard Châtillon	**Position:**	Neurosurgeon, CIUSSS-MCQ
**Name:**	Julie O'Shaughnessy	**Position:**	Clinician professor, UQTR
**Name:**	Charles Tétreau	**Position:**	Clinician professor, UQTR

Project Information

**Title of Study:**	The effect of chiropractor-informed triage versus usual medical management on low back pain patient outcomes and trajectories in tertiary care settings: a controlled clinical study.

**Keywords (seven maximum)**	Low back pain; triage; care pathways; chiropractor; pain; disability; health resources

**Total Amount requested:**	$ 50 000

**Institution or organization where research will be conducted**:	Université du Québec à Trois-Rivières

A letter indicating funding support from other entities will be included (if applicable). A letter verifying other funding will be required prior to CCRF funds being made available.

YES ☒ NO ☐

FINANCIAL OFFICER

Please provide the name and contact information of the financial officer who administers research funds at your Institution.

**Cheque Payable To:**	Université du Québec à Trois-Rivières
**Financial Officer:**	Caroline Béland
**Title:**	Research fund administrator
**Institution:**	Université du Québec à Trois-Rivières
**Mailing Address:**	3351, boul. des Forges, Trois-Rivières (Québec) G8Z 4M3<br>1051A, Pavillon Pierre Boucher
**E-mail:**	Caroline.Beland@uqtr.ca
**Telephone:**	819-376-5011, ext 2324
This information is required if funds are to be released to your institution	

ETHICAL APPROVAL

Please attach letter of ethics approval to the appendix, if available and applicable. If approval has not been received by the application deadline then evidence of approval must be received by CCRF no later than **90 business days** following grant approval**.**

☐ Ethics approval attached ☒ Letter to follow ☐ N/A

1. SUMMARY OF THE PROJECT:

Almost everyone will have low back pain at some point in their lives. Low back pain is a complex multifactorial condition for which diagnosis and clinical management remains a challenge. Factors such as wait times, delays in diagnosis or proper referral can result in Canadian patients having difficulty getting the care that they need.

The overall objective of this project is to explore how chiropractors, who specialize in the diagnosis and clinical management of spinal conditions, can transform healthcare trajectories and improve the health of patients with low back pain by integrating medical specialist team.

To do so, patients with low back pain seeking medical care within the public health system will be first seen by chiropractors. Chiropractors will play a key role in identifying the type of low back pain and subsequently offering guidance to physicians with regard to the best treatment and management options that are currently recommended. Participating patients will be followed over a year while extensive health-related data will be collected and compared to non-triage patients with low back pain.

This study will not only assess the impact of triaging on patients' clinical management and care trajectory but will be the first to extensively study the value of integrating chiropractors in the public healthcare system.

Offering tailored care to patients' suffering from low back pain within an interdisciplinary environment has been identified as a key priority to improve patients' health outcomes. The addition of chiropractors to usual medical care should improve patients' trajectories and healthcare resources use. Our approach is not only unique but inline with both the ***Health systems*** and ***Population Health*** national research priorities identified by CCRF.

2. ACCESS TO RESEARCH SITE & SUPPORT

The CIUSSS-MCQ has granted support to our research team which will benefit from the guidance of key personnel from the medical and clinical research service, namely its Head of service, Eva Mathieu, and its Scientific director, Julie Houle, during the implementation phase of the study.

Ethics certification and study progress will be monitored through *Nagano*, an online system acquired by CIUSSS-MCQ to oversee and manage research initiatives conducted on the premises. Marie-Claude Lehoux, clinical research coordinator at CIUSSS-MCQ, will provide assistance in meeting the institution convenience requirements.

The neurosurgery outpatient clinic affiliated to the CIUSSS-MCQ will serve as the recruitment site for the proposed study. The research team will be supported by the 5 neurosurgeons working onsite. The clinic will provide secretarial assistance for the identification of potential participants (using chief complaint reported at the time of appointment scheduling).

A research nurse, Joany Rousseau-Bédard, will conduct the data extraction from the participants' hospital electronic clinical record and the Quebec Health Record, for which access is limited to authorized health and social services professionals.

The conduct of the study requires minimum equipment that will be made available onsite prior to the start of recruitment. One chiropractic tables, readily available, will be lend by UQTR's chiropractic Department for the duration of the study to conduct the physical assessment. Smaller material, including reflex hammers and pinwheels (already available), desk computers, filing cabinets, and paper forms will be provided by the *Chaire de recherche internationale en santé neuromusculosquelettique*.

The members of the research team will have a privileged access to a working space within the outpatient clinic to ensure participants eligibility and provide study-related information, as well as a secure environment for storing research data. In addition, the research team will have access to a meeting room located at the regional hospital main site which has been made available to the personnel working within the *Chaire de recherche internationale en santé neuromusculosquelettique.* The fact that this meeting room is within the physicians' working environment, combined with the proximity of the research team members, will facilitate exchanges and progress meetings throughout the course of the study.

Letters of collaboration attached as an appendix: Yes ☒ No ☐ N/A ☐

3. SUMMARY OF RESEARCH PROPOSAL (ABSTRACT)

Scope: According to several studies, patients and society most often perceive chiropractors as ***spine experts*** who can efficiently assess spinal conditions and provide nonsurgical, non-pharmacological spine care. Nevertheless, they are scarcely integrated and have minimal interactions with public health systems and resources. Given that care seeking of patients with low back pain within the public health system often initiates a cascade of interventions that may not represent the most appropriate management for the patient, strategies involving focused implementation of best practice, analysis, and subsequent redesign of clinical pathways, are now almost unanimously recommended in national and clinical professional guidelines. One opportunity to improve patient care pathways is to assure that the first healthcare provider seen is best able to identify the patient's condition (or refer properly) and provide the treatment most likely to benefit a given individual. Given their training and field expertise, chiropractors could play a significant role in triaging and stratifying patients prior to medical consultations.
Purpose: The goals of this project are to [1] evaluate the effects of chiropractor-informed triage of patients with LBP on clinical outcomes and patients' trajectories, and [2] to study the extent of these effects within the tertiary care setting while describing and categorizing common care pathways. We hypothesise that a chiropractor-informed triage will result in equivalent patients' clinical outcomes but improved trajectories, including lesser use of diagnostic tests, total number of medical visits and unwarranted referrals to medical specialists compared to usual medical management.
Methodology: One hundred and forty consecutive patients scheduled in neurosurgery with a primary complaint of low back pain will be recruited to participate in this study. Patients will be randomized to either chiropractor-informed triage or usual medical management. The chiropractor-informed triage will consist of a 2-step process. Following the history taking and a physical exam, patients will be categorized into non-specific low back pain, radicular syndrome, or specific spinal pathology and subsequently stratified into low, moderate or high risk of poor prognosis using the STarT Back Screening Tool. Those with radicular syndrome or specific LBP will be assessed for referral needs. The working diagnosis and risk stratification/referral need will then be transferred to the neurosurgeon for only half of the group (n=70), based on the randomisation sequence. The neurosurgeons will in turn perform their own patient evaluation and decide on the best management strategy. It is believed that the extensive prognosis assessment provided by the chiropractor, which would otherwise not be routinely performed by neurosurgeons because of time and patient load demands, could influence their clinical management decisions. To measure the effects of chiropractor-informed triage, patient-reported outcomes (pain intensity, function, perceived change in the condition, quality of life, use of non-publicly funded care, expectation of recovery, and satisfaction) will be collected at baseline and at 1 week, 3, 6, and 12 months follow-up. In addition, the use of diagnostic tests and subsequent care visits will be monitored through the patients' chart over a one-year period following inclusion. General trends and assessment of outcomes variability over time will be explored using independent linear mixed model with repeated measures for group comparisons. A secondary analysis will provide information regarding the concordance between chiropractors and neurosurgeons decisions.
Anticipated impact: This project may provide the necessary preliminary data to help make the case for larger scale implementation of chiropractor-informed triage within the public health system. It advances knowledge and understanding of evidence-based management of patients with low back pain. Considering that several clinician-scientists will play leading roles in the project, two of its long-term goals are to provide unique capacity-building opportunities for future chiropractors to train in an interdisciplinary setting and foster the integration of the profession in the public health arena.

4. SEARCH PROPOSAL

PROBLEM STATEMENT AND LITERATURE REVIEW

Low back pain burden and low value care

Public healthcare systems are constantly under pressure with increasing costs, demands and limited resources. In all Canadian provinces, public healthcare represents the single largest budget item, ranging from roughly 35% percent of the total budget in Quebec to almost 45% percent in Ontario. Over the past 20 years, the share of government spending represented by healthcare and the ratio of healthcare spending relative to the size of the economy have consistently increased and led the Fraser Institute to describe the provincial governments increase in healthcare spending as “unsustainable”(1).

Chronic diseases and conditions are largely responsible for increasing costs in addition to causing substantial disability. Preventing the limitations associated to chronic conditions has now become one of the World Health Organization (WHO) top priorities (2). Indeed, the WHO considers that addressing people’s disability should help minimize the health, social and economic impacts of chronic health conditions, and improve well-being. A recent Canadian study exploring the burden of chronic illness trends concluded that, in the past ten years, arthritis, back pain, mood disorders, stroke and chronic obstructive diseases constantly have higher burden on health outcomes and health-related quality of life compared with other chronic conditions (3). Among the many chronic diseases, low back pain (LBP) and neck pain rank among the top five disorders with regard to years lived with disability, and the related expenses increase every year (4). According to the 2016 Global Burden of Disease Study, Disability-Adjusted Life Years (DALYs) associated with low back and neck pain have risen every decade, with an estimated increase of about 30 million people affected between 1990 and 2016. Not only does LBP have a significant burden on Canadians’ quality of life but access to public healthcare resources can be challenging and delays in referral from general practitioners to specialists are frequent (4).

In a recent “Call for action” published in the Lancet journal, LBP experts have proposed several initiatives to meet the challenges associated with prevention of disabling LBP (5). Among the proposed “actions”, changes in health systems include [1] *the development of clear care pathways and referral, to enable people to see the right person for delivery of the right treatment at the right time, while precluding use of alternative inappropriate pathways*, [2] *the development of consistent evidence-based clinical care standards and key indicators integrated across health-care systems and settings* and [3] *the development and implementation of cost-effective strategies that provide access to effective care for all.*

In the majority of healthcare systems from developed western countries, primary care physicians, often referred to as general practitioners (GPs), are the primary contacts responsible for referring patients with musculoskeletal (MSK) conditions to secondary and tertiary care and sometimes to other healthcare professionals (6). However, evidence suggests that the quality of care offered by physicians to treat MSK conditions is not optimal and that their diagnoses are often inaccurate (7). Furthermore, the usual care provided by physicians for LBP too often does not match the care endorsed in evidence-based guidelines and may delay or dismiss evidence-based treatment options affecting patients’ outcomes (7, 8). Unfortunately, this situation has not improved over time and recent studies still report usual care by GPs that include overuse of imaging, opioid prescriptions and underuse of advice and education (9).

High value care and other professions

In order to increase access to timely and more efficient care for MSK conditions, organizational innovations in care pathways have been proposed and evaluated in many countries (10). Such innovations include the involvement of other professions that specialize in MSK disorders. A recent systematic review assessing the impact of direct access physiotherapy compared to GPs’ usual medical care for patients with MSK disorders showed that MSK conditions management by physiotherapists yielded more valid

diagnoses, better clinical outcomes, more efficient use of resources, and diminished healthcare costs than GPs-led usual medical care (10). A recent systematic review concluded that, although models for MSK triage have shown positive results for early physiotherapeutic involvement, most studies were nonrandomized and only explored selection accuracy, waiting times for healthcare professional and patient satisfaction (10-12). While physiotherapy may have led the way in the field of MSK disorders care pathway innovations, we strongly believe that given their training and expertise, chiropractors can play a significant role in the management of spinal conditions within the Canadian public health system. This research question has yet to be answered and warrants careful and valid investigation.

Chiropractors' unique perspective and opportunities

Chiropractic has been struggling with its identity throughout most of its existence and several organizations and leaders of the profession have, in the past decades, advocated for a modernization of the profession's identity. Many leaders of the profession have suggested an identity that is shaped not only by chiropractors' specialized knowledge and training, but also supported by societal and patient needs and, of course, the best available scientific evidence. According to several studies, patients, health professionals and society most often perceive chiropractors as ***spine experts*** who can efficiently assess spinal conditions and provide nonsurgical, non-pharmacological spine care (13). Although publicly perceived as spine experts, chiropractors are not well integrated and have minimal interactions with public health systems and resources. A recent study showed that patients from Ontario chiropractors were most commonly referred from other patients and close relatives and that only 6.8% of encounters were referrals from a family physician (14). Chiropractors' involvement in public health structures and care pathways would most likely contribute to increase collaboration and integration of the profession.

Given that care seeking by patients with LBP within the public health system often initiates a cascade of interventions that may not represent the most appropriate management for the patient, strategies involving focused implementation of best practice, analyses and subsequent redesign of clinical care pathways are now almost unanimously recommended in national and professional guidelines (15). In fact, changes in clinical systems and pathways for patients with LBP are required and triaging and stratification approaches are among the most common recommended approaches in recent LBP evidence-based guidelines (7, 8).

This project is based on the premise that chiropractors can play an important role in the public health system care pathway of patients with LBP (16). Given their training and field expertise, chiropractors could play a significant role in triaging and stratifying patients prior to tertiary care medical consultations. Triaging of patients by chiropractors may play a significant role in reducing low-value care while supporting medical specialists. Triaging will provide key prognostic indicators for spinal conditions, potentially reducing inappropriate referrals leading to unnecessary use of specialised healthcare resources and longer waiting times.

RESEARCH OBJECTIVES AND HYPOTHESES

The goals of this project are [1] to evaluate the effects of chiropractor-informed triage of patients with LBP on clinical outcomes and patients' trajectories, and [2] to study the extent of these effects within the tertiary care setting while describing and categorizing common care pathways for LBP patients within the context of Canadian public healthcare. We hypothesize that a chiropractor-informed triage will result in equivalent patients' clinical outcomes but improved trajectories, including lesser use of diagnostic tests, decreased number of medical visits and improvement of referral patterns to medical specialists compared to usual practice.

METHODS AND PROCEDURES

Design and Recruitment sites

We propose a controlled clinical trial involving individuals seeking medical consultation in the tertiary care public health system for an episode of LBP. The study will be conducted at the neurosurgery outpatient clinic affiliated to the Centre Intégré Universitaire de Santé et de Services Sociaux- Mauricie Centre du Québec (CIUSSS-MCQ; Trois-Rivières' regional healthcare organization). The CIUSSS-MCQ, established in 2015 as a result of the merger of 12 health and social services public institutions, totals 974 physicians, including 515 GPs. It serves a population of over 510,000 inhabitants, of which 83% are over 18 years of age (17).

Eligibility

Patients were identified by the neurosurgery department's administrative staff at the time of appointment scheduling based on the primary complaint listed on the referral request. Patients who provided consent were subsequently contacted by a research assistant to ascertain their eligibility and received further information about the study- Inclusion criteria include being ≥18 years of age, being able to comprehend and express oneself in French, and presenting with pain consistent with current LBP definition that is pain between the lower rib margins and the buttock creases (18), with or without associated symptoms in the lower limb(s). Informed written consent will be obtained the day of the initial appointment. Patient's involved in litigation related to their LBP condition (Worker's compensation, public automobile insurance plan or other litigations) will be excluded from the study.

Sample and Randomization

One hundred and forty patients will be randomised to either chiropractor-informed triage or usual medical management. The sample size calculation is based on an equivalence study design, intended to demonstrate that the new approach (chiropractor-informed group) will lead to equivalent clinical outcomes compared to usual care (not chiropractor-informed group). The primary outcome is back pain intensity on a 0-10 numeric rating scale at 3 months. Equivalence is defined as the two-sided 95% confidence interval for the between-group mean difference (chiropractor-informed minus usual care) lying entirely within −1.2 to +1.2 points (equivalence margin Δ=1.2). Assuming a common SD of 2.0 points and a true mean difference of 0, a 1:1 allocation with the two one-sided tests (TOST) procedure (α=0.025 per one-sided test) required n = 80 participants per group to achieve 80% power. Allowing 15% attrition, we will recruit 70 participants per group (N=140). Randomization sequence will be computer-generated using blocks of 10, concealed using sequentially numbered, opaque sealed envelopes. The study protocol does not allocate the treatment each patient will receive, but rather if the specialist receives information following the chiropractor-informed triage process. Figure 1 illustrates enrollment of participants, randomization as well as setting and time points for outcome assessment.

Triaging process and Outcome assessment timeline

The chiropractor-led triage will consist of a 2-step process: **[1]** Following a standardized history taking and a physical examination, **all patients** will be categorized into non-specific LBP, radicular syndrome, or specific spinal pathology (see table 1 for clinical key features). **[2]** Participants will be subsequently stratified into levels of risk of poor prognosis using the STarT Back Screening Tool (SBST). The SBST is a brief prognostic screening tool that guides LBP management. The questionnaire categorizes psychosocial risk for levels of pain, disability and distress as low, medium or high (19, 20). Risk categories are used to inform management strategies. Those diagnosed with radicular syndrome or specific LBP will subsequently be stratified into one of the 3 subcategories addressing referral needs (i.e. wait and see, referral to a medical specialist/further tests needed, or medical emergency) Figure 2 illustrates the two-

step triaging process. In correspondence to each of the 2 triaging steps, basic information on the rationale for chiropractor-informed triage will be shared with the participating neurosurgeons at the beginning of the study. Following the chiropractor triage assessment, the working diagnosis and risk stratification or referral need will be communicated to the neurosurgeon using a standardized clinical note, prior to the patient's consultation and without further discussion for only half the group (n=70). The neurosurgeon will in turn perform its own patient evaluation and decide on the best management strategy. It is believed that the extensive prognosis assessment provided by the chiropractor, which would otherwise not be routinely performed by tertiary care physicians because of time and patient load demands, could influence their clinical management decisions.

To measure the effects of chiropractor-led triage, patient-reported outcomes will be collected at baseline and at 1 week, 3, 6, and 12 months follow-up. In addition, patients' trajectories of healthcare utilization will be monitored over a one-year period following inclusion in the trial (see figure 1).

Instrument reliability and validity

Low back clinical outcomes

Low back clinical outcomes will be tracked using validated and reliable self-reported outcome measures. Current and mean (past week) ***level of pain intensity*** for back and leg pain will be measured using an 11-point numerical rating scale (NRS) which is a global measure of pain intensity anchored by two extremes of pain intensity ranging from 0 ('no pain') to 10 ('pain as bad as it could be'). It has good short-term test-retest reliability, good construct validity and can distinguish levels of pain in subjects with chronic pain (21-23). ***Function*** will be measured with the Oswestry Disability Index (ODI) which regroups ten questions related to different domains including pain intensity, personal care, lifting, walking, sitting, standing, sleeping, sexual life, social life, and traveling. Each question is rated on a scale of 0 to 5 points with a maximum score of 50. Higher scores indicate greater disability (25). The French version of the ODI is a valid, reliable, and responsive condition-specific assessment tool that is suited for use in clinical practice (26). ***Health-related quality of life*** will be measured using the WHO Quality of Life-BREF questionnaire, which assesses the individual's perceptions in the context of their culture and value systems, and their personal goals, standards and concerns. The instrument comprises 26 items, which measure the following four domains: physical health, psychological health, social relationships, and environment (27). Higher scores denote higher quality of life. General ***expectation of recovery*** will be measured using a 11-point NRS for which the patients will be asked the following question: How likely do you think it is that you will have a complete recovery? Possible answers ranging from -5= "very unlikely" to +5="very likely", with 0 being "I don't know" (28). ***Perceived Global Rating of Change*** in the condition will be measured using the following question: *With respect to your low back pain, how would you describe yourself now compared to the initial onset of symptoms*? Participants will select between one of the seven following choices: 1) completely better; 2) much improved; 3) slightly improved; 4) no change; 5) slightly worse; 6) much worse and 7) worse than ever (29). ***Patient satisfaction*** with the initial outpatient visit will be measured using items 5 to 9 from the Visit-Specific Satisfaction Instrument (VSQ9) (30). Patients will be asked to rate their experience with both the chiropractor and physician on a 5-point Likert scale (0=poor to 5=excellent) for each of the following items: 5-Time spent with the healthcare professionals you saw; 6-Explanation of what was done for you; 7-Technical skills (thoroughness, carefulness, competence) of the healthcare professionals you saw; 8-The personal (courtesy, respect, sensitivity, friendliness) of the person you saw; 9-The visit overall.

Patients' trajectory outcomes

The ***diagnosis and clinical management*** (i.e. self-management; including advice or brief intervention/medication), referral to other first line care practitioner (chiropractor, physiotherapist, etc.)

or medical specialist, injection, leave of absence, etc.) decided by the physician will be retrieved immediately after the initial encounter and compared to the chiropractor's working diagnosis, risk stratification or referral need according to current evidenced-bases practice guidelines. Requests and results (if patients follow recommendations) of ***medical imaging*** and ***laboratory tests*** produced by an institution or a private medical laboratory in the province of Quebec, as well as ***medication prescriptions*** that are dispensed in community pharmacies will be monitored through the patient's Quebec Health Record (QHR). ***Subsequent visits*** to the outpatient clinic will be monitored (including date of visit, type of professional, number of visit, prescribe medication, referrals) through the patient's electronic clinical record (ECR), which is used in all health and social services across the CIUSSS-MCQ institutions. The ***use of non-publicly funded care*** (including any care that was fully paid out of pocket or reimbursed by a personal insurance coverage) will be tracked by asking patients at follow-up if they sought first line care for their current LBP episode since the last assessment time point. Type of practitioner, timing and number of visits attended and whether the patient was discharged from care will be recorded.

Data analyses plan

For the equivalence hypothesis, between-group comparisons of back pain outcomes at the primary endpoint (3 months) will be conducted using independent-samples *t* tests on pain change scores. Equivalence will be evaluated using the TOST procedure, with equivalence margins set at ±1.2 points on the NRS-11. Equivalence will be concluded when the 95% confidence interval of the difference in pain change scores (Δ intervention − Δ control) is entirely contained within the predefined equivalence bounds. General trends and assessment of outcome variability over time will be explored using independent linear mixed model with repeated measures for group comparisons. In addition, in order to characterize the patterns of change in low back outcome (back pain as the main outcome) occurring in patients over the one year follow-up period, a latent class growth analysis will be performed using SAS Proc Traj (31). A secondary analysis will rely on the definition of a taxonomy of the trajectories of care. We will first categorize the variables describing the patients' pathways according to patients' trajectory outcomes. We will then search for relationships between these variables using multiple correspondence analysis (32) followed by ascending hierarchical classification to built a taxonomy of the trajectories and classify each participants according to these patterns of healthcare use. Trajectories will be compared by cross-tabulation with triage groups, and other demographic and clinical variables, to test the effect of triage on the improvement of care trajectories.

ETHICAL CONSIDERATIONS

Ethical certification will be obtained from both UQTR and CIUSSS-MCQ ethical review boards for human research who already have a reciprocity agreement facilitating the realization of clinical research involving UQTR's researchers and students in collaboration with CIUSSS-MCQ. All aspects of the project will be evaluated using the principles and norms proposed by the *Tri-Council Policy Statement: Ethical Conduct for Research Involving Humans* – TCPS 2 (33). Written informed consent will be obtained from all participants according to the same guiding principles prior to their inclusion in the study.

RELEVANCE TO CCRF AND CONTRIBUTION TO MUSCULOSKELETAL HEALTH

The proposed research project is the first stage of an ambitious research program investigating the potential impact of chiropractors' integration in MSK public health services. We believe that the proposed study investigating the effects of chiropractor-led triage on healthcare use and clinical outcomes in patients with LBP is relevant to both the ***Health systems*** and ***Population Health*** national research priorities identified by CCRF. The results of our study will not only increase knowledge regarding the potential effects of chiropractic inclusion within the public healthcare realm but also raise awareness about the unique training and expertise that chiropractors can bring to the field of MSK health. Taking

appropriate actions to prevent chronicity, limiting surgery and ensuring efficient delivery of high-quality primary, secondary, tertiary care may require the integration of paramedical MSK health professionals. We are convinced that chiropractors can play a key role in meeting this public health priority. Therefore, triaging by chiropractors as active members of an interdisciplinary healthcare strategy within the public health system across Canada may not only play a critical role in the care of patients with low back pain by improving patient outcomes, but also by reducing overall healthcare costs by minimizing specialist referrals, expensive imaging and unproven pharmacological treatments. Our approach will lay the foundation for integration of chiropractic care for other MSK conditions.

ROLE OF PI, CO-PIS AND OTHER KEY PERSONNEL

Dr Descarreaux and **Dr Marchand** have been leading research initiatives in collaboration with CIUSSS-MCQ medical staff and research personnel for the past five years. They both will be responsible for overseeing the methodological aspects of the conduct of the study and ensuring compliance with institutional regulations. Dr Descarreaux will lead the project within the tertiary care setting with Dr Marchand. Graduate students will work under the supervision of Drs Descarreaux and Marchand as they will be enrolled in Master and Doctorate health-related programs offered at UQTR. **Dr. Hincapié's** research interests are to study the etiology, prognosis and treatment of MSK disorders. His areas of expertise include randomized clinical trials, chiropractic health care quality improvement, implementation science, and descriptive, analytical, and translational epidemiology. He has contributed to the design of the trial and will be a key contributors to graduate student supervision and data analyses. **Dr Schweinhardt**, Head of Research at the Department of Chiropractic Medicine at the University of Zurich, has extensive expertise in pain research with various patient populations. More recently, she has contributed to and led cohort studies in back pain patients that are similar in design to the proposed study. **Dr Beauséjour** specializes in epidemiology of health care utilization and the study of care pathways of patients with MSK complaints from primary to specialized care. In the current project, she will coordinate data analysis for the identification and characterization of patient outcome trends and care trajectories. **Dr Châtillon** is chief of the neurosurgery service at CIUSSS-MCQ. His practice comprises primarily of spinal surgical pathologies requiring instrumented and non-instrumented surgery. He has a research interest in non-surgical and pre-surgical management of neuromusculoskeletal disorders. He has been involved in the study's design and will oversee patient recruitment at the neurosurgery outpatient clinic. **Dr O'Shaughnessy** oversees the junior internship at the university-based outpatient chiropractic teaching clinic. Since 2006, in collaboration with our research group, she has conducted and implanted eleven different clinical studies within the teaching clinic. She will play a key role in training and mobilising research interns, clinicians, and other key staff throughout the research program. **Professor Tétreau** is the director of UQTR's outpatient Kinesiology Clinic. Being involved in several project with the neurosurgery team from CIUSSS-MCQ, professor Tétreau will play an important role in the coordination of the trial within the tertiary care setting. **Dr Brousseau-Foley,** a close collaborator, is a general physician at the CIUSSS-MCQ Family Medicine Group clinic. She will be responsible for promoting the research project among her peers while playing a pivotal role in the coordination between the medical and the research teams. Although the amended version of the project does not include primary care triaging Dr Brousseau-Foley will play an important role in understanding the impact of tertiary care triaging on general physicians' management of non-specific low back pain.

PLANS FOR KNOWLEDGE TRANSLATION

Although our project main goal is not to develop knowledge translation tools in the first stage of our research program (year 1-3), several clinicians and chiropractic students will be involved in the study. The project will provide unique capacity-building opportunities for future chiropractors to train in an interdisciplinary setting and foster the integration of the profession in the public health arena. In addition

to scientific communications, the training workshops developed for chiropractors, physicians and students involved in the project will be used as a stepping stone in the implementation of a formal chiropractic and medical student training module on LBP management.

FIGURES, TABLES AND REFERENCES

Figure 1: Study flowchart

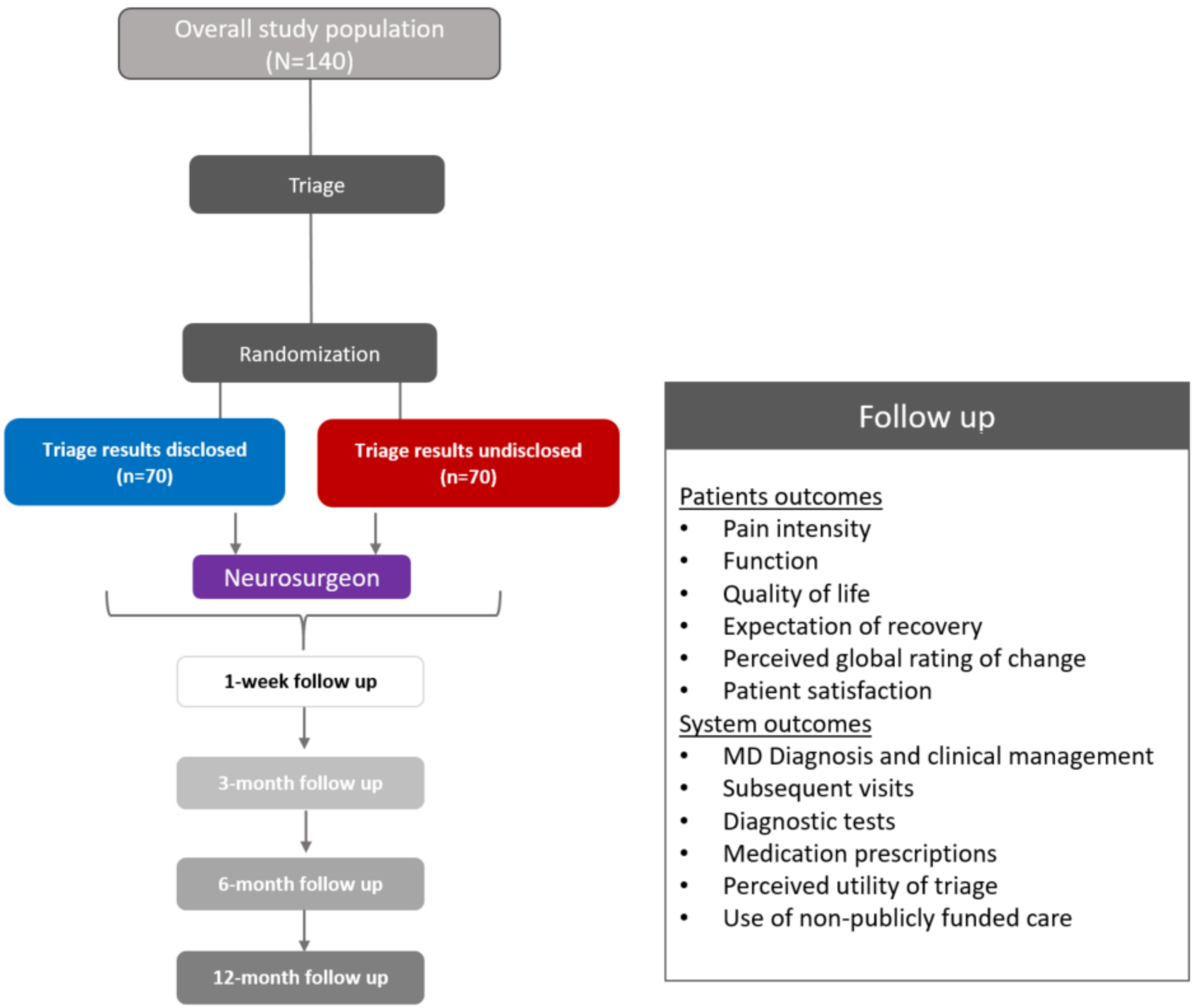

Figures 2: Study flowchart

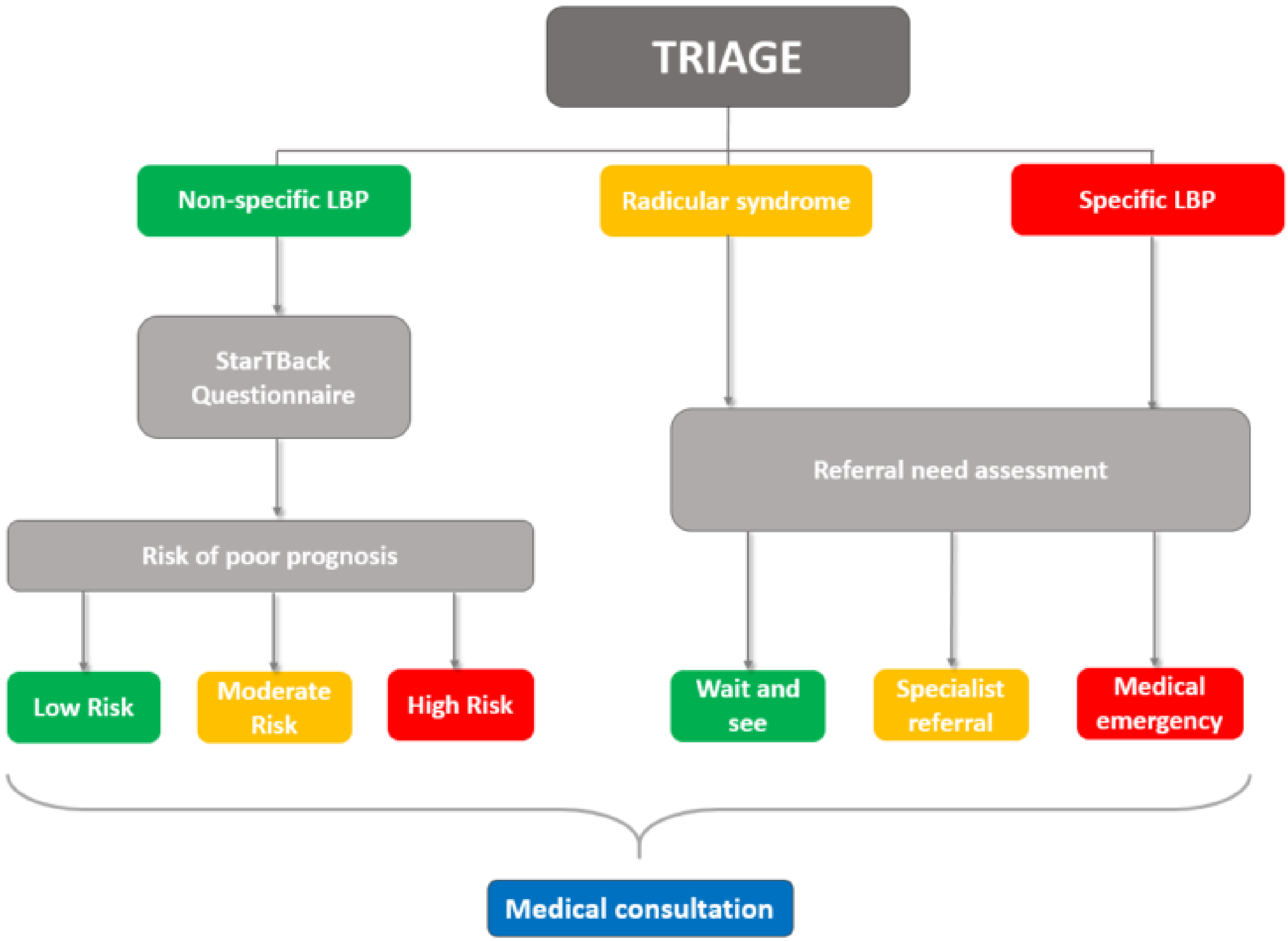

Figure 3: timeline for the project

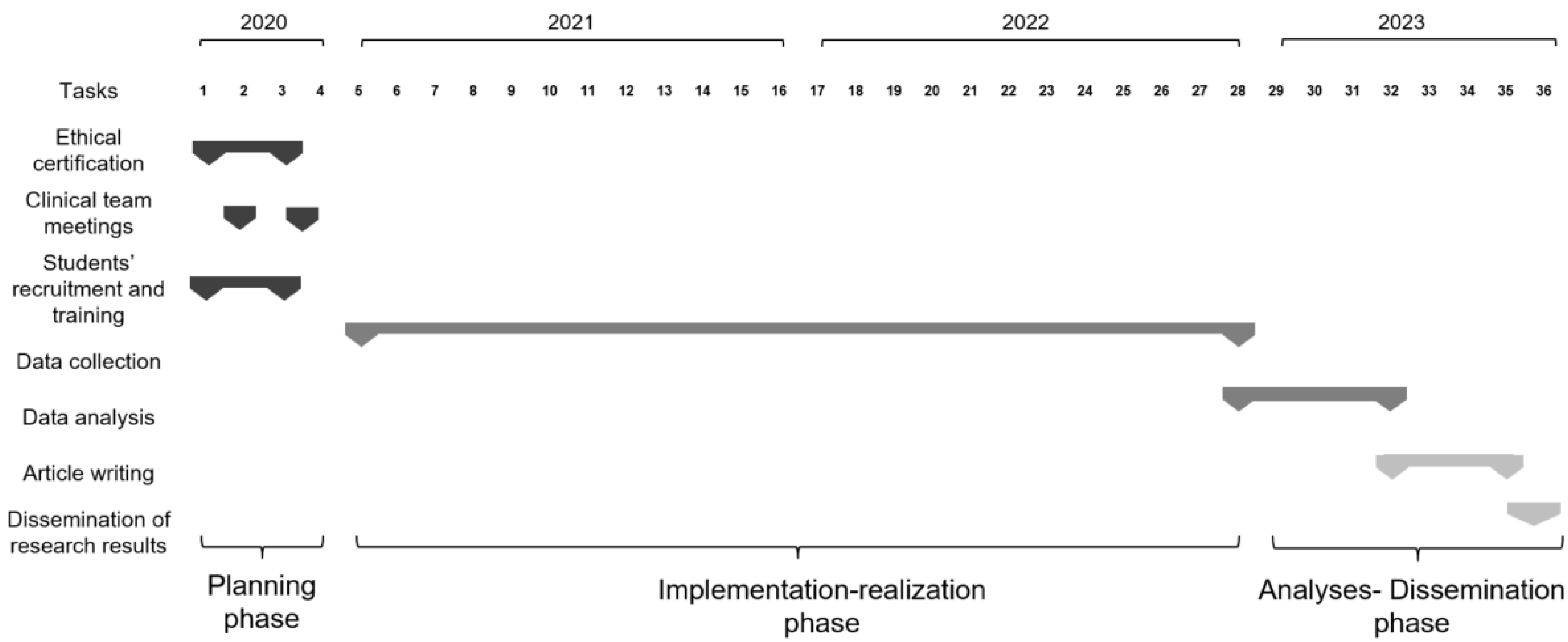

Tables 1: Characteristics and diagnostic features of LBP types

Non-Specific LBP	Radicular syndrome			Specific LBP	
Exclusion of radicular syndrome and specific LBP<br>No identifying features	**Pathology**	**Characteristics**	**Physical examination**	**Pathology**	**Characteristics**
	**Radicular pain**	- Leg pain typically worse than back pain<br>- Leg pain quality (sharp, lancinating or deep ache increasing with cough, sneeze or strain)<br>- Leg pain location (unilateral, dermatomal concentration) (below knee for L4, L5, S1)	• Positive provocative tests for dural irritation: straight leg raise (L4, L5, S1, S2) and prone knee bend (L2, L3, L4)<br>• Lumbar extension and ipsilateral side flexion may exacerbate radicular pain (Kemp sign)<br>• Sometimes accompanying radiculopathy signs	**Vertebral fracture**	- Older age (> 65 years for men, > 75 for women)<br>- Prolonged corticosteroid use<br>- Severe trauma<br>- Presence of contusion or abrasion
	**Radiculopathy**	- Numbness or paraesthesia (typically in distal dermatome)<br>- Weakness or loss of function (eg, footdrop)	***Sensory:*** diminished light touch or pinprick in dermatomal distribution, paraesthesia intensifies with lumbar extension<br>***Motor:*** myotomal weakness<br>***Reflexes:*** reduced or absent knee jerk or ankle jerk	**Malignancy**	- History of malignancy<br>- Strong clinical suspicion<br>- Unexplained weight loss, >50 years
	**Lumbar spinal stenosis**	- Neurogenic claudication limiting walking tolerance<br>- Older patient, bilateral leg pain or cramping with or without LBP<br>- Bilateral leg pain exacerbated by extended	• Normal neurological assessment during rest (sometimes mild motor weakness or sensory changes)<br>• Antalgic postures (stooped standing and walking), straightened	**Spinal Infection**	- Fever or Chills<br>- Immune compromised patient<br>- Pain at rest or at night<br>- IV drug user<br>- Recent injury, dental or spine procedure

		posture (eg, standing) and relieved by flexion (eg, sitting, bending forward and recumbent posture)	posture can amplify leg pain or numbness<br>• Wide based gait	**Axial spondylo-arthritis**	Chronic back pain (> 3 months' duration), with back pain onset before 45 years of age and one or more of the following:<br><br>- Inflammatory back pain (at least four of: age at onset 40 years or younger, insidious onset, improvement with exercise, no improvement with rest, and pain at night — with improvement when getting up);<br>- Peripheral manifestations (in particular arthritis, enthesitis or dactylitis);<br>- Extra-articular manifestation (psoriasis, inflammatory bowel disease or uveitis);<br>- Positive family history of spondyloarthritis; and<br>- Good response to non-steroidal anti-inflammatory drugs
				**Cauda equina syndrome**	- New bowel or bladder dysfunction<br>- Perineal numbness or saddle anaesthesia<br>- Persistent or progressive lower motor neuron changes

REFERENCES

1. Bacchus Barua MPaJE. The Sustainability of Health Care Spending in Canada 2017. Fraser institute. 2017.
2. (WHO) WHO. Rehabilitation: key for health in the 21st century. 2017;Rehabilitation 2030: A Call for Action.
3. Usuba K, Li AKC, Nowrouzi-Kia B. Trend of the burden of chronic illnesses: using the Canadian Community Health Survey. Public Health. 2019;177:10-8.
4. B. B. Waiting Your Turn - Wait times for Health Care in Canada. 2017.
5. Buchbinder R, van Tulder M, Öberg B, Costa LM, Woolf A, Schoene M, et al. Low back pain: a call for action. Lancet. 2018;391(10137):2384-8.
6. Bury TJ, Stokes EK. Direct access and patient/client self-referral to physiotherapy: a review of contemporary practice within the European Union. Physiotherapy. 2013;99(4):285-91.
7. Williams CM, Maher CG, Hancock MJ, McAuley JH, McLachlan AJ, Britt H, et al. Low back pain and best practice care: A survey of general practice physicians. Arch Intern Med. 2010;170(3):271-7.
8. Bishop PB, Wing PC. Compliance with clinical practice guidelines in family physicians managing worker's compensation board patients with acute lower back pain. Spine J. 2003;3(6):442-50.
9. Kamper SJ, Logan G, Copsey B, Thompson J, Machado GC, Abdel-Shaheed C, et al. What is usual care for low back pain? A systematic review of health care provided to patients with low back pain in family practice and emergency departments. Pain. 2020;161(4):694-702.
10. Demont A, Bourmaud A, Kechichian A, Desmeules F. The impact of direct access physiotherapy compared to primary care physician led usual care for patients with musculoskeletal disorders: a systematic review of the literature. Disabil Rehabil. 2019:1-12.
11. McEvoy C, Wiles L, Bernhardsson S, Grimmer K. Triage for Patients with Spinal Complaints: A Systematic Review of the Literature. Physiother Res Int. 2017;22(1).
12. Marks D, Comans T, Bisset L, Scuffham PA. Substitution of doctors with physiotherapists in the management of common musculoskeletal disorders: a systematic review. Physiotherapy. 2017;103(4):341-51.
13. Schneider M, Murphy D, Hartvigsen J. Spine Care as a Framework for the Chiropractic Identity. J Chiropr Humanit. 2016;23(1):14-21.
14. Mior S, Wong J, Sutton D, Beliveau PJ, Bussières A, Hogg-Johnson S, et al. Understanding patient profiles and characteristics of current chiropractic practice: a cross-sectional Ontario Chiropractic Observation and Analysis STudy (O-COAST). BMJ open. 2019;9(8):e029851.
15. Foster NE, Anema JR, Cherkin D, Chou R, Cohen SP, Gross DP, et al. Prevention and treatment of low back pain: evidence, challenges, and promising directions. Lancet. 2018;391(10137):2368-83.
16. Garner MJ, Birmingham M, Aker P, Moher D, Balon J, Keenan D, et al. Developing integrative primary healthcare delivery: adding a chiropractor to the team. Explore. 2008;4(1):18-24.
17. CIUSSSMCQ. Rapport annuel de gestion. 2019.
18. Dionne CE, Dunn KM, Croft PR, Nachemson AL, Buchbinder R, Walker BF, et al. A consensus approach toward the standardization of back pain definitions for use in prevalence studies. Spine (Phila Pa 1976). 2008;33(1):95-103.
19. Bruyère O, Demoulin M, Beaudart C, Hill JC, Maquet D, Genevay S, et al. Validity and reliability of the French version of the STarT Back screening tool for patients with low back pain. Spine (Phila Pa 1976). 2014;39(2):E123-8.
20. Bruyère O, Demoulin M, Brereton C, Humblet F, Flynn D, Hill JC, et al. Translation validation of a new back pain screening questionnaire (the STarT Back Screening Tool) in French. Arch Public Health. 2012;70(1):12.
21. Hawker GA, Mian S, Kendzerska T, French M. Measures of adult pain: Visual Analog Scale for

Pain (VAS Pain), Numeric Rating Scale for Pain (NRS Pain), McGill Pain Questionnaire (MPQ), Short-Form McGill Pain Questionnaire (SF-MPQ), Chronic Pain Grade Scale (CPGS), Short Form-36 Bodily Pain Scale (SF-36 BPS), and Measure of Intermittent and Constant Osteoarthritis Pain (ICOAP). Arthritis Care Res (Hoboken). 2011;63 Suppl 11:S240-52.
22. Jensen MP, Karoly P, Braver S. The measurement of clinical pain intensity: a comparison of six methods. Pain. 1986;27(1):117-26.
23. Wewers ME, Lowe NK. A critical review of visual analogue scales in the measurement of clinical phenomena. Res Nurs Health. 1990;13(4):227-36.
24. de Vet HC, Heymans MW, Dunn KM, Pope DP, van der Beek AJ, Macfarlane GJ, et al. Episodes of low back pain: a proposal for uniform definitions to be used in research. Spine (Phila Pa 1976). 2002;27(21):2409-16.
25. Vogler D, Paillex R, Norberg M, de Goumoëns P, Cabri J. [Cross-cultural validation of the Oswestry disability index in French]. Ann Readapt Med Phys. 2008;51(5):379-85.
26. Vianin M. Psychometric properties and clinical usefulness of the Oswestry Disability Index. J Chiropr Med. 2008;7(4):161-3.
27. Skevington SM, Lotfy M, O'Connell KA. The World Health Organization's WHOQOL-BREF quality of life assessment: psychometric properties and results of the international field trial. A report from the WHOQOL group. Qual Life Res. 2004;13(2):299-310.
28. Cormier S, Lavigne GL, Choinière M, Rainville P. Expectations predict chronic pain treatment outcomes. Pain. 2016;157(2):329-38.
29. Kamper SJ, Maher CG, Mackay G. Global rating of change scales: a review of strengths and weaknesses and considerations for design. J Man Manip Ther. 2009;17(3):163-70.
30. Rubin HR, Gandek B, Rogers WH, Kosinski M, McHorney CA, Ware JE, Jr. Patients' ratings of outpatient visits in different practice settings. Results from the Medical Outcomes Study. JAMA. 1993;270(7):835-40.
31. Jones BL, Nagin DS, Roeder K. A SAS procedure based on mixture models for estimating developmental trajectories. Sociological methods & research. 2001;29(3):374-93.
32. Lebart L, Morineau A, Piron M. Statistique exploratoire multidimensionnelle: Dunod Paris; 1995.
33. Canadian Institutes of Health Research NSaERCoC, and Social Sciences and Humanities Research, Council. Tri-Council Policy Statement : Ethical Conduct for Research Involving Humans. 2018;RR4-2/2019E-PDF.
