# Supplementary material for: Effects of musculoskeletal expert input on low back pain outcomes: A randomised controlled trial
Source: PLoS One. 2026 Aug 26;21(8):e0356604. doi: 10.1371/journal.pone.0356604 (PMC13513973; doi:10.1371/journal.pone.0356604)
Supplement: Supplementary file 3 [file pone.0356604.s003.pdf]

Santé
et Services sociaux
Québec

DT9430

ADULT NEUROSURGERY CONSULTATION

Patient's first and last name		
Health insurance number	Expiry Year	Month
Parent's first and last name		
Area code Phone number	Area code Phone number (alt.)	
Address		
Postal code		

Note:

1- For clinical alerts and priorities A and B (refer on the back of the form) communicate directly with the neurosurgeon on call.
2- Notify the patient to bring a CD-ROM copy of the radiological imaging for the appointment with the specialist.
3- Check the reason for consultation and complete the mandatory section on suspected diagnosis and clinical information.
4- Attach other relevant investigation reports if available.

Clinical priority scale: B : ≤ 10 days C : ≤ 28 days D : ≤ 3 months E : ≤ 12 months

For priority A consultations (≤ 3 days), do not send them to the CRDS; use the following corridors: specialist on call, accueil clinique, etc.

Reason for consultation

Category	Reason	Option	Priority
Spine cervico-dorsal	☐ Compressive myelopathy with symptoms > 8 weeks *(Prerequisite: MRI report < 12 months and other investigation reports)*		C
Spine cervico-dorsal	Painful or sensory-motor radiculopathy *(Prerequisite: MRI report < 12 months and other investigation reports) (e.g. cervical disc herniation)*	☐ With severe symptoms and functional limitations (ADLs/DA) > 8 weeks	D
Spine cervico-dorsal		☐ With moderate chronic symptoms > 8 weeks	E
Spine cervico-dorsal	☐ Neck pain/Thoracic spine pain **with anatomical instability**, seen on imaging, without spinal cord or nerve root involvement *(Prerequisite: imaging report and other investigation reports if available)*		D
Spine lumbo sacral	Painful or sensory-motor radiculopathy or neurogenic claudication *(Prerequisite: MRI report < 12 months and other investigation reports) Unsuccessful 8 weeks trial of physical therapy and medication*	☐ With severe symptoms and functional limitations (ADLs/DA) > 8 weeks	D
Spine lumbo sacral		☐ With moderate chronic symptoms > 8 weeks	E
Spine lumbo sacral	☐ Isolated low back pain with structural abnormality (scoliosis, spondylolysis, spondylolisthesis, spinal stenosis, foraminal stenosis) *(Prerequisite: MRI report < 12 months and other investigation reports)*		E
Peripheral nerves	Compressive neuropathy (e.g. carpal tunnel or cubital tunnel) *(Prerequisite: EMG < 1 year and other investigation reports)*	☐ With motor deficit	D
Peripheral nerves		☐ Without motor deficit	E
Peripheral nerves	☐ Peripheral nerve tumor *(Prerequisite: MRI or ultrasound < 12 months)*		D
Tumor	☐ Extraparenchymal intracranial tumors: meningioma, schwannoma, pituitary tumor, skull or skull base tumor *(Prerequisite: MRI report or CT scan and other investigation reports)*	☐ With progressive neurological symptom	C
Tumor		☐ Without progressive neurological symptom	D
Vascular	☐ Cerebral aneurysm, arterio-venous malformation, dural fistula, cavernoma (without hemorrhage) *(Prerequisite: MRI report or CT scan and other investigation reports)*		D
Vascular	☐ Asymptomatic carotid stenosis (≥70%) *(Prerequisite: imaging report and other investigation reports)*		D
Functional	☐ Craniofacial neuralgia (e.g. trigeminal) refractory to medical therapy *(Prerequisite: MRI report and other investigation reports)*		D
Functional	☐ Neuromodulation for chronic pain syndrome or for spasticity *(Prerequisite: investigation reports)*		E
Cranial miscellaneous	☐ Chronic hydrocephalus or normal pressure hydrocephalus *(Prerequisite: imaging report and other investigation reports)*		D
Cranial miscellaneous	☐ Intracranial cyst (e.g. arachnoid, pineal gland) *(Prerequisite: MRI report or CT scan and other investigation reports)*		E
Cranial miscellaneous	☐ Cranial lesion with benign features (e.g. cyst, bone malformation) *(Prerequisite: MRI report or CT scan and other investigation reports)*		E
Cranial miscellaneous	☐ Type 1 Chiari malformation : symptomatic1 or with a syrinx		D

☐ **Other reason for consultation or clinical priority modification** ***(MANDATORY justification in the next section):*** — **Clinical priority**

Suspected diagnosis and clinical information (mandatory)

If prerequisite is needed :

☐ **Available in the QHR**
☐ **Attached to this form**

Special needs:

Referring physician identification and point of service

Referring physician's name		Licence no.
Area code Phone no.	Extension	Area code Fax no.
Name of point of service		

Stamp

Signature — Date (year, month, day)

Family physician: ☐ Same as referring physician ☐ Patient with no family physician

Family physician's name

Name of point of service

Registered referral (if required)

If you would like a referral for a particular physician or point of service

Clinical alerts and priority A or B (non-exhaustive list)
**Communicate with the neurosurgeon on call**
• Intracranial hemorrhage<br>• Syndrome of intracranial hypertension with or without alteration of consciousness<br>• Sudden or rapidly progressive onset of neurological deficit (arising from brain, spinal cord, cauda equina or acute radiculopathy with motor deficit)<br>• Acute or subacute myelopathy (spinal cord compression) with rapid evolution of symptoms<br>• Symptomatic carotid stenosis<br>• Acute moderate or severe cranio-cerebral or spinal cord traumatic injury<br>• Cranial or spinal fracture<br>• Intracerebral brain tumors: metastasis, gliomas or others<br>• Intradural or extradural spinal tumors (primary or metastatic)

Legend

[1] Associated symptoms with Type 1 Chiari malformation are the following : headache during exercise, difficulty swallowing, sleep apnea. At least one of these symptoms is required.
