# Supplementary material for: Effects of musculoskeletal expert input on low back pain outcomes: A randomised controlled trial
Source: PLoS One. 2026 Aug 26;21(8):e0356604. doi: 10.1371/journal.pone.0356604 (PMC13513973; doi:10.1371/journal.pone.0356604)
Supplement: Supplementary file 4 [file pone.0356604.s005.pdf]

Clinical note

Last name, First Name : Date of the assessment :

Date of birth :

Clinical profile

Dominant site of pain : ☐ Back ☐ Leg (___________)

The pain is : ☐constant ☐ intermittent

Physical examination

Sensory function : (____________________________________)
Motor function : (_____________________________________)
Reflexes : (____)

Pathological reflexes : (___________________________________)

Level of disability (Oswestry)

☐ minimal ☐ moderate ☐ severe ☐ crippled ☐ bed-bound or exaggerating symptoms

Risk of poor prognosis (StarT Back)

☐ minimal ☐ moderate ☐ severe

Diagnosis and management strategy

☐ Non-specific low back pain ☐ Radicular syndrome ☐ Specific low back pain ☐ Other causes

☐ Conservative management ☐ Neurosurgery referral indicated ☐ Further diagnostic tests needed

☐ Falls under another specialty ☐ Medical emergency

Signature
