# Supplementary material for: Effects of musculoskeletal expert input on low back pain outcomes: A randomised controlled trial
Source: PLoS One. 2026 Aug 26;21(8):e0356604. doi: 10.1371/journal.pone.0356604 (PMC13513973; doi:10.1371/journal.pone.0356604)
Supplement: Supplementary file 5 [file pone.0356604.s004.docx]

**Variables used to inform diagnostic and management strategies.**

| Category | Variable | Outcome |
| --- | --- | --- |
| Clinical data | | |
| History taking | Presence of red flags (i.e., indicators of an underlying pathology) | Bladder and bowel dysfunction, saddle anesthesia, fever, chills, progressive neurological symptoms, history of trauma, prolonged corticosteroid use, recent history of infection, previous history of cancer, unexplained weight loss, intravenous drug use, being immunocompromised, morning stiffness, improvement in back pain with exercise but not with rest, awakening because of pain in the second half on the night, alternating buttock pain, and failure to improve with conservative care |
|  | Pain dominance | Back, leg(s) or both |
|  | Pain status | Intermittent, constant |
|  | Pain duration | Acute (< 4 wks), Subacute (4-12wks) or chronic (> 12 wks) |
|  | Symptoms progression | Stable, aggravating, improving |
|  | Prognostic factors | History of spinal surgery  Smoking status  Type and number of comorbidities |
| Self-reported questionnaires | Pain intensity | NRS-11: measure of pain intensity ranging from 0 (no pain) to 10 (pain as bad as it could be) |
|  | Disability | ODI: regroups 10 questions related to different domains including pain intensity, personal care, lifting, walking, sitting, standing, sleeping, sexual life, social life, and traveling. Each question is rated on a scale of 0 to 5 points with a maximum score of 50. Higher scores indicate greater disability. |
|  | Risk of poor prognosis | SBST: brief validated tool (9-item version): designed to screen patients with LBP for prognostic factors and group patients in 3 categories of risk of poor outcome – low, medium, and high risk |
| Physical examination | Presence of neurological deficits | Sensory, motor, deep tendon reflex deficits |
|  | CNS disorder | Presence of pathological reflexes |
| Care trajectories data | | |
| History taking | Use of advanced imaging and diagnostic testing for LBP prior to the neurosurgery consult | Number and type |
|  | Use of conservative care prior to the neurosurgery consult | Number and type of treatments  Type of health professional seen |

CNS: Central nervous system; NRS-11: 11-point Numerical Rating Scale; ODI: Oswestry Disability Index; SBST: STarT Back Screening Tool; wks: weeks
